# Accelerated Adaptive Evolution on a Newly Formed X Chromosome

**DOI:** 10.1371/journal.pbio.1000082

**Published:** 2009-04-14

**Authors:** Doris Bachtrog, Jeffrey D Jensen, Zhi Zhang

**Affiliations:** 1 Department of Integrative Biology, University of California, Berkeley, Berkeley, California, United States of America; 2 Division of Biological Sciences, University of California, San Diego, La Jolla, California, United States of America; University of Bath, United Kingdom

## Abstract

Sex chromosomes originated from ordinary autosomes, and their evolution is characterized by continuous gene loss from the ancestral Y chromosome. Here, we document a new feature of sex chromosome evolution: bursts of adaptive fixations on a newly formed X chromosome. Taking advantage of the recently formed neo-X chromosome of Drosophila miranda, we compare patterns of DNA sequence variation at genes located on the neo-X to genes on the ancestral X chromosome. This contrast allows us to draw inferences of selection on a newly formed X chromosome relative to background levels of adaptation in the genome while controlling for demographic effects. Chromosome-wide synonymous diversity on the neo-X is reduced 2-fold relative to the ancestral X, as expected under recent and recurrent directional selection. Several statistical tests employing various features of the data consistently identify 10%–15% of neo-X genes as targets of recent adaptive evolution but only 1%–3% of genes on the ancestral X. In addition, both the rate of adaptation and the fitness effects of adaptive substitutions are estimated to be roughly an order of magnitude higher for neo-X genes relative to genes on the ancestral X. Thus, newly formed X chromosomes are not passive players in the evolutionary process of sex chromosome differentiation, but respond adaptively to both their sex-biased transmission and to Y chromosome degeneration, possibly through demasculinization of their gene content and the evolution of dosage compensation.

## Author Summary

Sex chromosomes have evolved independently many times in both animals and plants from ordinary chromosomes. Much research on sex chromosome evolution has focused on the degeneration and loss of genes from the Y chromosome. Here, we describe another principle of sex chromosome evolution: bursts of adaptive fixations on a newly formed X chromosome. By employing a comparative population genomics approach and taking advantage of the recently formed sex chromosomes in the fruit fly Drosophila miranda, we show that rates of adaptation are increased about 10-fold on a newly formed X chromosome relative to background levels of selection in the genome. This suggests that a young X chromosome responds adaptively to both its female-biased transmission and to Y chromosome degeneration. Thus, contrary to the traditional view of being passive players, the X chromosome has a very active role in the evolutionary process of sex chromosome differentiation.

## Abstract 3

Research on sex chromosome molecular evolution has focused on the degeneration of the Y chromosome, but new evidence highlights that important changes occur on the evolving X chromosome in the form of rapid bursts of adaptive evolution.

## Introduction

Sex chromosomes have originated independently many times in both animals and plants from ordinary autosomes [1,2]. Their evolution is characterized by a loss of gene function on the nonrecombining Y chromosome, as seen in many taxa [3–5]. For example, of the roughly 1,000 genes originally present on the ancestral Y chromosome of humans, only a few dozen remain [3]. Conventionally, X chromosomes were often viewed as static entities in the evolutionary process of sex chromosome differentiation, with relatively little change occurring that would distinguish the X from the autosome from which it was derived [2]. However, several recent studies have shown that the X chromosome has also undergone substantial evolutionary modifications (reviewed in [6–9]).

In particular, genes on X chromosomes are faced with several unusual challenges relative to autosomal genes. First, the degeneration of the Y chromosome creates a gene dose problem for X-linked genes in males [10], resulting in the evolution of dosage compensation mechanisms on the X [10–13]. Another consequence of Y chromosome degeneration is the hemizygosity of X-linked genes in males, increasing the efficacy of natural selection acting on recessive mutations (known as faster-X evolution [14]). Finally, sex-biased transmission of X chromosomes can result in an accumulation, or deficiency, of genes with female- or male-beneficial functions [15–17]. Indeed, ancestral X chromosomes have often evolved dosage compensation mechanisms, and male-specific genes are depleted (i.e., demasculinization of the X chromosome; [11,12,17]). Genes on a newly formed X chromosome may therefore undergo accelerated evolutionary change relative to background levels of adaptation in the genome, to adjust to their altered genomic environment [13,18].

**Figure 1:**
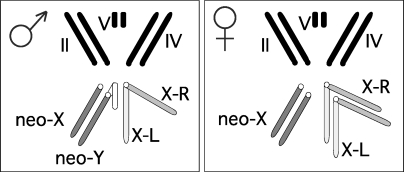
Karyotype of Drosophila miranda The ancestral X chromosome consists of two chromosomal arms; X-L (light grey), which is part of the X chromosome in all species of the genus *Drosophila* (>60 MY old), and X-R (medium grey), a former autosome that fused to X-L approximately 10 MY ago. The neo-sex chromosomes (dark grey) were formed by the fusion of another autosome to the ancestral Y chromosome about 1 MY ago. The neo-X chromosome segregates with the X chromosome in D. miranda, but is not fused to it. X-R has already acquired all the stereotypical properties of X chromosomes, whereas the neo-sex chromosomes are in transition from an ordinary autosome to a pair of heteromorphic sex chromosomes.

To test for signatures of pervasive adaptive evolution at the DNA level on a newly formed X chromosome, we take advantage of the unusual sex chromosomes (termed neo-sex chromosomes) of Drosophila miranda (Figure 1). In the genus *Drosophila*, fusions between autosomes and the ancestral sex chromosomes (that is, the original X and Y chromosomes shared by all members of the genus *Drosophila*) have repeatedly created so-called neo-sex chromosomes [19–21]. As a result of such a fusion, one chromosome—the neo-Y—is cotransmitted with the Y chromosome through males only. Given the lack of crossing over in male *Drosophila*, such fusions restrict recombination between the male-limited neo-Y chromosome and its former homolog (the neo-X chromosome). In fact, the neo-Y chromosome is completely sheltered from recombination and thus exposed to the evolutionary forces causing Y degeneration [19–21]. The neo-X chromosome, in contrast, can still recombine in females and cosegregates with the ancestral X chromosome (i.e., it is present in two copies in females and one copy in males). Over evolutionary time periods, neo-sex chromosomes of several *Drosophila* species have evolved the classical properties of ancestral sex chromosomes (i.e., the neo-Y chromosome degenerates, and the neo-X chromosome evolves dosage compensation [19–21]).

The ancestral X chromosome of D. miranda consists of two arms (Figure 1); X-L (Muller's element A), which is part of the X chromosome in all species of the genus *Drosophila* (and >60 million years [MY] old, [22]), and X-R (Muller's element D), which became part of the X only approximately 10 MY ago, and this X-autosome fusion is shared by species in the D. affinis and D. pseudoobscura subgroup [11,12]. Interestingly, X-R has already acquired the classical characteristics of an evolved X chromosome, including the evolution of dosage compensation over all its length [11,12] and demasculinization of its gene content [17]. The neo-sex chromosomes of D. miranda (Muller's element C) were formed about 1 MY ago (∼10*N*_e_ generations) [23], and appear to be in transition from an ordinary autosome to a pair of heteromorphic sex chromosomes [4]. Specifically, about half of all genes have become pseudogenized on the neo-Y chromosome of D. miranda [24], and some genes on the neo-X are acquiring dosage compensation [11,12].

Here, we describe patterns of DNA sequence polymorphism at many gene fragments across the D. miranda neo-X chromosome and compare them to gene fragments surveyed from the ancestral X chromosome. Contrasting patterns of polymorphism of genes from the recently formed neo-X with genes located on the ancestral X allows us to control, to some extent, for recent demographic events and life-history differences that otherwise pose a problem for identifying adaptive evolution using population variability data [25–27]. Thus, the unusual chromosomal configuration of D. miranda enables us to test for an elevation in rates of adaptation on a recently formed X chromosome relative to background rates of adaptive evolution in the genome [28].

## Results and Discussion

### Reduced Diversity on the Neo-X

Natural selection can increase the frequency of a beneficial mutation in a population, thereby reducing neutral variation in the genomic region linked to the advantageous allele (i.e., a selective sweep [29,30]). Thus, one signature of directional selection at the DNA level is a reduction in neutral variation in genomic regions surrounding the targets of selection [29]. To test for increased rates of adaptation on the D. miranda neo-X chromosome, we surveyed DNA sequence polymorphism at 152 gene fragments across the neo-X and compare them to 112 gene fragments from the ancestral X chromosome (60 genes from X-L and 52 from X-R). Certain classes of genes tend to undergo increased rates of adaptive evolution in *Drosophila*, such as genes showing sex-biased expression or genes involved in some biological pathways [31,32]. Genes on both the neo-X and the ancestral X chromosome were selected randomly with regard to gene function or expression patterns, and no significant heterogeneity in gene count among gene ontology classes or patterns of sex-biased expression was detected between loci on the ancestral X chromosome and the neo-X (see Tables S1–S4).

Estimated levels of synonymous variation and synonymous divergence to an outgroup species are similar for genes on X-L and X-R (mean *π*_s_ = 0.51% vs. *π*_s_ = 0.71%; Wilcoxon two-sample test, *p* > 0.05 and mean *K*_s_ = 4.26% vs. *K*_s_ = 3.98% between D. miranda and D. pseudoobscura; Wilcoxon two-sample test, *p* > 0.05), consistent with observations that X-R has reached the typical properties of an evolved X chromosome (i.e., X-R appears fully dosage compensated in males and its gene content shows a similar deficiency of male-biased genes as X-L, the ancestral X chromosome; [12,17]). Table 1 summarizes average levels of synonymous diversity across the genomic regions studied. Synonymous site diversity is reduced by about 50% on the neo-X compared to the ancestral X (average *π*_s_ = 0.33% vs. *π*_s_ = 0.60%; Wilcoxon two-sample test, *p* < 3e-6), while levels of synonymous divergence to an outgroup species between the chromosomes are similar (*K*_s_ = 4.36% vs. *K*_s_ = 4.13% for the neo-X and the ancestral X chromosome between D. miranda and D. pseudoobscura; Wilcoxon two-sample test, *p* = 0.13), suggesting that the two chromosomes have similar mutation rates. Comparison of polymorphism and divergence levels at synonymous sites on the neo-X versus the ancestral X using a Hudson-Kreitman-Aguadé (HKA) test [30] confirms that the reduced diversity observed at neo-X genes is not attributable to a lower mutation rate (HKA test *p* < 10^−4^, see Table S6). Additionally, many more invariant loci are observed on the neo-X chromosome (23 vs. three genes, Table 1).

**Table 1:**
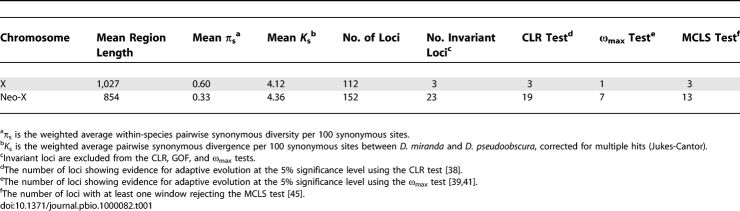
Average Diversity Measures in Drosophila miranda across X-Linked and Neo-X Gene Fragments and Numbers of Loci Showing Evidence for Recent Adaptive Evolution

Nonequilibrium demography, such as recent population bottlenecks, or differences in life-history strategies between males and females can cause levels of diversity to differ between sex chromosomes and autosomes [25–27]. However, because the ancestral X chromosome and the neo-X chromosome show identical patterns of inheritance, demography and life history are expected to influence patterns of diversity on the X and the neo-X in a similar manner [25–27]. Note, however, that the neo-X chromosome was segregating as an autosome until the formation of the neo-sex chromosomes roughly 1 MY ago. This event was presumably associated with a modest decline in the population size of the neo-X (from 2*N* to 1.5*N*), but is sufficiently ancient (∼10*N*_e_ generations ago) to not leave signatures in current levels of population variation [33,34]. Thus, the ancestral X should serve as an adequate control for demographic effects on the neo-X, which suggests that natural selection is responsible for reduced levels of variability on the neo-X relative to the ancestral X. We employed several statistical approaches in order to quantify rates of adaptive evolution on the neo-X versus the ancestral X.

### More Recent Selective Sweeps on the Neo-X

Recent positive selection results not only in a local reduction of variation in the genomic region surrounding the target of selection, but also in a skew in the frequency distribution of mutations surrounding the target of selection (i.e., the hitchhiking effect; [29,35]). In particular, recent adaptive evolution in the genome results in an excess of both low- and high-frequency mutations relative to neutral expectations [33,36,37]. Such approaches to detect selection using population variability data cannot be applied to invariant loci, but the 129/152 polymorphic neo-X–linked and 109/112 polymorphic X-linked loci can be examined.

A composite likelihood ratio (CLR) test [38] that utilizes these population variation patterns to detect recent adaptations reveals that a greater proportion of genes located on the neo-X chromosome relative to the ancestral X reject a model of neutral sequence evolution in favor of a genetic hitchhiking model at the 5% significance level (19/129 testable neo-X genes, 15%, vs. 3/109 testable X-linked genes, 3%, Table 1; *p* = 0.0013, Fisher exact test). Further, the distributions of CLR test *p*-values for the neo-X and X are significantly different from one another (*p* = 2e^−9^, Kolmogorov-Smirnov test). Although the CLR test is not robust to some demographic scenarios [34], the ancestral X chromosome functions as an internal control to account for such effects (see above). Thus, we detect about 5-fold more adaptive events on the newly formed neo-X compared to the ancestral X. To further evaluate evidence in support of more adaptation on the neo-X, we also applied a goodness-of-fit test (GOF test) [34] to genes rejecting the CLR test. This statistic was proposed to assess the fit of data to a selective sweep model, in order to identify loci with significant CLR tests that may be explained by demographic effects. Only one of the three genes that rejected the CLR test on the ancestral X chromosome were consistent with a selective sweep model using the GOF test, whereas 13 of the 19 neo-X genes rejecting the CLR test were consistent with a recent selective sweep (Table 1).

Adaptive evolution also leaves characteristic signatures in patterns of linkage disequilibrium (LD), with reduced LD across the target of selection, and increased LD in genomic regions flanking the target [39–41]. The ω_max_ statistic [39] was used to identify loci under selection based on these patterns of LD. Again we detect more selection on the neo-X chromosome (7/129 testable genes, 5%, reject neutrality at the 5% significance level) compared to the ancestral X (1/109 testable genes, 1%, reject neutrality at the 5% significance level, Table 1; *p* = 0.07 Fisher exact test). The genes identified as targets of recent adaptive evolution using the ω_max_ statistics are a subset of those identified with the CLR+GOF test. Thus, several locus-by-locus tests identify a much larger fraction of genes having undergone recent adaptive evolution on the neo-X chromosome compared to the ancestral X.

Note that the locus-by-locus tests for selection are not corrected for multiple testing, because our main interest lies in quantifying the relative excess of statistical tests rejecting neutrality on the neo-X relative to the ancestral X, and not the absolute number of significant rejections. A similar excess of adaptive evolution of neo-X–linked genes relative to the ancestral X is found if we use the false discovery rate to account for multiple testing (see Table S7). In addition, the above locus-by-locus tests of selection assume that the genomic regions surveyed are unlinked. Indeed, the genes surveyed on the neo-X and the ancestral X chromosome appear mostly independent from each other, with levels of LD being similarly low between loci (unpublished data). Also, the genomic regions we identified as having undergone recent selection on the neo-X show little evidence of clustering (the median distance between loci using the D. pseudoobscura genome sequence as a guide is 0.97 Mb for the 19 significant loci identified using the CLR test, and only two regions rejecting this test are adjacent to each other). In addition, many of the gene fragments studied here were mapped previously by in situ hybridization experiments, and found to be scattered along the polytene chromosomes of D. miranda [42–44]. Thus, most selective sweeps identified on the neo-X represent independent events.

**Figure 2:**
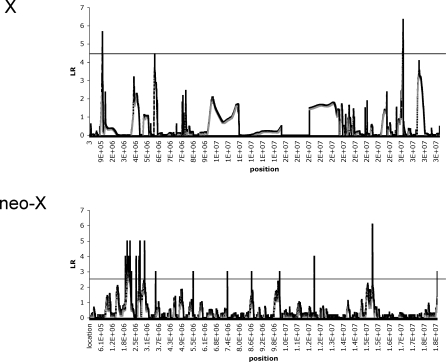
The Maximized Composite Likelihood Surface Calculated for Genes from the Ancestral X and the Neo-X Chromosome The horizontal line indicates the 5% cutoff values (LR_crit_) as determined separately for the X (LR_crit_ = 4.5) and the neo-X (LR_crit_ = 2.7) by simulation under a neutral equilibrium model. More significant peaks are identified on the neo-X, suggesting that more recent selective sweeps have occurred on this chromosome.

A more formal approach to take account of multiple testing and linkage when comparing rates of evolution on the ancestral and the newly formed X chromosome is to consider all loci simultaneously rather than testing them individually. To this end, we employed a composite likelihood method (the maximized composite likelihood surface test [MCLS test]) [45] for detecting positive selection, which uses a similar likelihood framework as the CLR test. As opposed to the CLR or GOF tests, however, which use a specific population genetic model as the null (the equilibrium neutral model or a selective sweep model, respectively), the MCLS statistic derives the null model from the empirical data itself. In this way, the test seeks to identify loci that show unusual patterns of variation relative to the other loci (the “background loci” [45]) in the genomic screen. To determine significance of the test statistic, it is still necessary to simulate data based on an explicit model [45]. In our implementation, we use the background allele frequency distribution (the background site-frequency spectrum) obtained from the ancestral X, to test for selection on both the ancestral X and the neo-X chromosome. Again, we find evidence for many more genomic regions having undergone recent positive selection on the neo-X (13 nonoverlapping regions) compared to the ancestral X (three genomic regions, Figure 2; *p* = 0.035, Fisher exact test). The distributions of the minimum *p*-value windows are significantly different for loci surveyed on the neo-X and the ancestral X (*p* = 0.022, Kolmogorov-Smirnov test). All 13 neo-X regions identified as targets of recent adaptive evolution using the MCLS approach correspond to loci identified as positively selected using the locus-by-locus CLR+GOF test (above).

Could other systematic biases—such as differences in overall recombination rates or levels of variability between sampled loci—result in differential power to detect selection on the two chromosomes? Specifically, selective sweeps are more easily identified in low-recombining regions due to the increased effects of hitchhiking [34], and statistical tests have reduced power to detect selection if levels of variability are low [34]. Recombination rates do not appear to systematically differ between loci on the neo-X and the ancestral X chromosome (average levels of LD, as measured by Wall's B or Q [46], are not significantly different within loci on the two chromosomes; *p* > 0.2, Kolmogorov-Smirnov test). However, the neo-X chromosome has significantly reduced levels of variability and more invariant genes relative to the X (Table 1), as expected under a model of recurrent selection. Methods based on the site-frequency spectrum (CLR, GOF, and MCLS) and LD (ω_max_) have reduced power to detect selection when levels of variability are low (and cannot be applied to invariant loci), implying less power to detect selective events on the neo-X chromosome. This suggests that the difference in rates of adaptive evolution between the neo-X and the ancestral X is likely underestimated. Nevertheless, utilizing many different features of our data, we consistently estimate that the fraction of genes having undergone recent adaptive evolution on the newly formed X chromosome increases 5–10-fold relative to background levels of adaptation on the ancestral X.

### Increased Rates of Adaptation on the Neo-X

**Figure 3:**
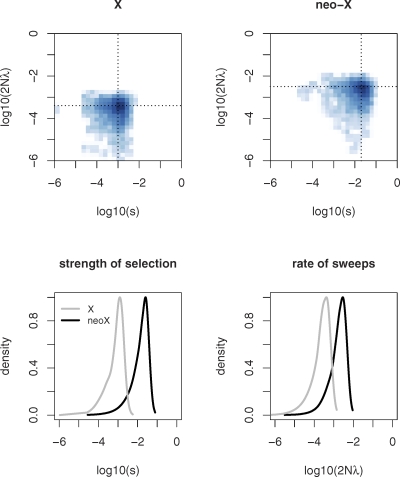
Approximate Bayesian Estimation of the Rate of Adaptive Substitutions (2*N_e_λ*) and Their Average Effect on Fitness (*s*) for Genes from the Ancestral X and the Neo-X Chromosome Estimation is based on 10^6^ draws from the prior (*s* ∼ Uniform (1.0E−06, 1.0) and 2*N_e_λ* ∼ Uniform (1.0E−07, 1.0E−01)). (Top) The joint posterior distributions for the X and neo-X. The dotted lines correspond to MAP estimates, and darker regions indicate greater posterior density. (Bottom) The marginal posterior distributions for the strength and rate of sweeps. The marginal posterior distribution for the X is indicated in grey, and for the neo-X in black. Both the rate and the strength of selection are inferred to be an order of magnitude higher for neo-X–linked genes.

To what extent are rates of adaptation accelerated on the neo-X? To evaluate the difference in rates of adaptation on the neo-X versus the ancestral X using all loci (including invariant ones), we estimate parameters of a recurrent selection model for each chromosome. Under a model of recurrent adaptation, the average reduction in levels of variability depends on the rate at which adaptive substitutions occur (2*N*_e_ *λ*) and their average effect on fitness (*s*) [47]. We use a recently developed approximate Bayesian approach [48] to estimate these parameters. This approach uses multiple summary statistics of the population variation data (see Materials and Methods), to obtain maximum a posteriori (MAP) estimates of both 2*N*_e_ *λ* and *s*. Figure 3 shows the marginal and joint posterior distributions of the rate and the strength of selection inferred for loci on the ancestral X and the neo-X. The MAP estimate of the rate of adaptive substitutions again is roughly 10-fold higher for neo-X–linked genes compared to genes that are located on the ancestral X (Figure 3). Interestingly, we also estimate the strength of selection to be an order of magnitude higher for genes on the neo-X chromosome (Figure 3). Importantly, MAP estimates of the strength and the rate of sweeps for the neo-X fall outside of the 95% credibility intervals for the estimates on the X. Thus, not only does adaptive evolution appear to be more frequent on a newly formed X chromosome, but also the selective benefit of mutations arising may be larger for genes that have only recently become X-linked relative to genes that have been evolving under a stable chromosomal configuration. This may be expected if genes on a young X chromosome are further away from their optimum fitness [49], since they experience a new genomic environment (i.e., they used to segregate as an autosome but have only recently become X-linked).

### Conclusions

Using a variety of approaches, we consistently infer a higher rate of adaptive evolution on the neo-X chromosome relative to the ancestral X. Systematic differences between the chromosomes, such as biases in the types of genes studied or overall differences in rates of recombination, appear unlikely to account for elevated rates of adaptation on the neo-X. Instead, increased adaptation is likely related to the recent formation of the X chromosome, and as such, there are a priori reasons to expect rapid adaptive changes to respond to novel selective pressures created by Y degeneration and sex-biased transmission. Specifically, old X chromosomes have evolved dosage compensation mechanisms [12], and genes with male-biased expression are underrepresented on the *Drosophila* X [17], resulting from selective gene extinction and gene movement off the X [17]. In addition, hemizygosity of X-linked genes in males increases the efficacy of natural selection acting on recessive mutations (faster-X evolution [14]). Expression profiling and comparative sequence analysis have shown both the acquisition of dosage compensation and demasculinization of gene content on chromosome element X-R in D. pseudoobscura (Muller's element D), a neo-X chromosome that formed approximately 10 MY ago [12,17]. We observe no evidence for elevated rates of adaptation on X-R relative to the older X-L (Muller's element A) in D. miranda, which is consistent with the functional data that X chromosomes evolve dosage compensation and demasculinization of their gene content in a relatively short evolutionary time period (i.e., within 10 MY). In contrast, the much younger neo-X chromosome in D. miranda (Muller's element C) appears still in transition from being an ordinary autosome to an X chromosome and exhibits chromosomal patterns intermediate between the autosomes and the ancestral X. In particular, the neo-X chromosome of D. miranda has evolved partial dosage compensation [11,12]. Here, we have shown that this evolutionary transition from an autosome to an X chromosome has been accompanied by a tremendous acceleration in rates of adaptive evolution, which presumably reflects the ongoing acquisition of dosage compensation on the neo-X of D. miranda [10] and possibly the demasculinization of its gene content. Thus, contrary to being a passive player in sex chromosome evolution, we demonstrate that the X chromosome has a very active role. We propose that a newly formed X chromosome actively responds to both its female-biased transmission and to Y degeneration, through bursts of adaptive evolution.

## Materials and Methods

### Survey of coding regions.

A total of 112 X-linked and 152 neo-X–linked gene fragments were surveyed in this study with a sample size of 12–18 D. miranda alleles (mean sample size 16). Genes were selected randomly with respect to gene function and expression patterns (see Tables S1–S4). The D. pseudoobscura genome sequence (release 2.0, http://flybase.org/) was used to provide estimates of divergence. Details of PCR primers are available from the authors on request. Information about the individual loci surveyed and the geographic origin of the D. miranda strains investigated can be found in Tables S1–S5. Many of the gene fragments studied were mapped previously to the polytene chromosomes of D. miranda using in situ hybridization, and found to be scattered along the ancestral X chromosome and the neo-X [42–44].

Standard PCR procedures were used to amplify each region from genomic DNA from single male flies, using X or neo-X specific primers. PCR products were cleaned using Exonuclease I and Shrimp Alkaline Phosphatase, and sequenced on both strands with the original PCR primers and internal sequencing primers if necessary, using Big-Dye (Version 3; Applied Biosystems). Sequence reactions were cleaned with sephadex plates (Edge Biosystems) and run on an ABI 3730 capillary sequencer. Chromatograms were edited and assembled using Sequencher (Gene Codes) software and multiple sequence alignments were generated using MUSCLE (http://www.drive5.com/muscle/) with protein-alignment–assisted adjustments to preserve reading frames. Exon–intron boundaries were determined from the D. pseudoobscura genome sequence annotation (release 2.0). Sequences have been deposited in GenBank under accession numbers FN252903–FN256223.

### Polymorphism and divergence analysis.

A library of Perl scripts was used to calculate the estimated number of synonymous sites, average pairwise diversity (*π*) and average pairwise divergence to D. pseudoobscura (*K*). A Jukes-Cantor correction was used to correct π and *K* for multiple hits. Insertion–deletion polymorphisms and polymorphic sites overlapping alignment gaps were excluded from the analysis. To compare polymorphism and divergence on the neo-X and the ancestral X, we implemented a multilocus two-class HKA test [30]. The 262 loci were pooled into two classes (i.e., X-linked vs. neo-X–linked). The *p*-values for this test are based on 10,000 replicate simulations of the standard neutral coalescent model using the program *ms* [50] with global (i.e., speciation time, *T*) and locus-specific parameters (i.e., sample size, *n*, and population mutation rate, *θ*).

### Locus-by-locus tests for selection.

Several statistical tests to identify recent adaptive evolution were applied to genes on the X versus the neo-X chromosome. The CLR test [38] uses the spatial distribution of mutation frequencies in a genomic region and levels of variability among a population sample of DNA sequences to test for evidence of a selective sweep. This method compares the ratio of the composite likelihood of the data under the standard neutral model of constant population size, neutral evolution, and random mating, *L_N_* (Data) to the composite likelihood of the data under the model of a selective sweep, *L_S_*(α̂,X̂|Data), where *α* is the maximum likelihood estimate (MLE) of 2*Ns*, and *X* is the MLE of the location of the beneficial mutation. The CLR test statistic employed is 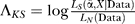 . The null distribution of Λ*_KS_* is obtained for each region by applying the CLR test to datasets obtained from simulation under the standard neutral model (using the program *ms* [50]) with the observed region length (*L*) and *θ*. The recombination rate ρ per site is set at 8.8 × 10^−8^ per site per generation [28]. For each locus, 1,000 neutral replicates were simulated using locus-specific parameters in order to assess significance. A complete users manual, as well as all necessary code, can be found at: http://www.yuseobkim.net/YuseobPrograms.html. The neutral model is rejected at level γ (5% used here) when the observed Λ*_KS_* is greater than the 100(1−γ) percentile of the null distribution.

The CLR test is sensitive to deviations from the assumptions of the standard neutral model, with population substructure and recent bottlenecks leading to a high false-positive rate [34]. However, demography is not a concern for our study as we only compare patterns on the neo-X to the ancestral X, which serves as an internal control for the inflated variances of the test statistics, associated with demographic effects. The ancestral X chromosome is an ideal control for that purpose, since it has the same population size and shows identical patterns of inheritance as the neo-X, and thus is influenced by demography and life history in a similar manner. To assesses the fit of individual loci to a selective sweep model, we also employed a GOF test that contrasts the null hypothesis *H_0_* that the data are drawn from a selection model as simulated by the CLR test to the alternative hypothesis *H_A_* that the data are not drawn from such a model [34]. A composite likelihood scheme is used to approximate the probability of the data given the null, *P*(Data|*H*_0_), to the probability of the data given the alternative, *P*(Data|*H*_A_), on the basis of the site frequency spectrum of mutations. Simulations (using the program *ssw* [38]) under the null hypothesis are used to find the critical value of the composite likelihood ratio GOF statistic for each region, with locus-specific (maximum likelihood) estimates of *S*, *L*, *α*, and *X*. Note that in this instance, the null model is a selective sweep as this test is employed conditional on rejecting the CLR test [34]. The program for calculating this statistic is available for download at http://www.yuseobkim.net/YuseobPrograms.html.

Positive selection results in strong LD flanking the target of selection, and reduced LD across the target [40,41]. We also employ patterns of LD to test for selection at individual loci using the *ω* test [39]. The *ω*-statistic, which is defined as

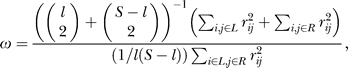
divides the *S* polymorphic sites in the dataset into two groups, one from the first to the *l*th polymorphic site from the left and the other from the (*l* + 1)th to the last site (*l* = 2, … , *S* − 2), where *L* and *R* represent the left and right set of polymorphic sites, and *r_ij_* ^2^ is the squared correlation coefficient between the *i*th and *j*th sites. Thus, *ω* increases with increasing LD within each of the two groups and decreasing LD between the two groups (i.e., the larger the value of the statistic, the more “sweep-like” the underlying pattern). For a locus, the value of *l* that maximizes *ω* (*ω*_max_) is found. Singletons were excluded prior to calculation. The null distribution of *ω* for each genomic region is obtained from simulation under the standard neutral model (using the program *ms* [50]) with fixed *θ* and *L*. As above, we set *ρ* = 8.8 × 10^−8^ per site per generation. The program for calculating this statistic is available for download at: http://www.molpopgen.org/software/libsequence.html.

### Multilocus tests for selection.

We used a multilocus test for selection based on a modification of the CLR test proposed by Nielsen et al. [45], called the MCLS test, to compare rates of adaptation on the ancestral X and the neo-X chromosome. Rather than using the standard neutral model to define the test statistic, this multilocus approach uses the background site frequency spectrum obtained from the data itself to test for selection. We use the site frequency spectrum of the ancestral X chromosome of D. miranda, to test for selection on both the ancestral X and the neo-X. This approach is not model independent, however, as coalescent simulations under the standard neutral model, using the exact configuration of the empirical dataset (with each locus having a unique *θ* and *L*), are necessary to determine the significance of the test statistic. This method takes polymorphism data and creates a grid of locations over the given region, and compares the maximum composite likelihood ratio of the hypothesis of a sweep with the null hypothesis of no sweep for each location. The parametric approach used is described by equation 6 of [45]. The gridsize parameter was set at 10^4^. As above, we set *ρ* = 8.8 × 10^−8^ per site per generation. We document the minimum *p*-value window for each surveyed locus. The program for calculating this statistic is available for download at: http://fisher.berkeley.edu/cteg/software.html.

To estimate selection parameters under a recurrent hitchhiking model, we use the approximate Bayesian approach of Jensen et al. [48]. The level of reduction in variation due to recurrent selection depends on the joint parameter 2*N_e_sλ* [47]. Both the rate, *λ*, and the fitness effect, *s*, of recurrent selection are estimated based upon their relationship with the means and standard deviations of common polymorphism summary statistics (the mean average pairwise diversity [*π*], the number of segregating sites [*S*], *θ_H_*, and *ZnS*; see [48]). Calculating these summary statistics from the observed data, and from simulated data with parameters drawn from uniform priors, we implement the regression approach of Beaumont et al. [51] which fits a local-linear regression of simulated parameter values to simulated summary statistics, and substitutes the observed statistics into a regression equation. The prior distributions used were *s* ∼ Uniform (1.0E−06, 1.0) and 2*N_e_λ* ∼ Uniform (1.0E−07, 1.0E−01), and the tolerance, *ɛ* = 0.001. Estimation is based on 10^6^ draws from the prior using the recurrent selective-sweep coalescent simulation machinery described in ref. [48]. We set *ρ* = 8.8 × 10^−8^ per site per generation and *N_e_* = 568,851 [28]. For inferences on selection parameters assuming an exponential distribution of *λ* and *s* see Figure S1. A complete users manual, as well as all necessary code for estimating *λ* and *s*, can be found at: http://www.molpopgen.org/software/JensenThorntonAndolfatto2008/.

Sequences have been deposited in GenBank under accession numbers FN252903–FN256223.

## Supporting Information

Figure S1 Estimating Selection Parameters Assuming an Exponential Distribution of the Rate and Strength of Selection Approximate Bayesian estimation of the rate of adaptive substitutions (2*N*λ) and their average effect on fitness (*s*) for genes on the ancestral X and the neo-X chromosome. Estimation is based on 10^6^ draws from the prior (*s* ∼ Uniform (1.0E−06, 1.0) and 2*N_e_* λ ∼ Uniform (1.0E−07, 1.0E−01)), where the selection parameters within a given replicate dataset are given by exponential distributions (see Materials and Methods for details on the inference procedure).
(A) The joint posterior distributions for the X and neo-X. The dotted lines correspond to MAP estimates, and darker regions indicate greater posterior density.
(B) The marginal posterior distribution for the X is indicated in blue, and for the neo-X in black.
(105 KB PDF)

Table S1 Locus-Specific Estimates of Population Parameters, Expression Bias, and Gene Function for Loci on the Ancestral X Chromosome (188 KB DOC)

Table S2 Locus-Specific Estimates of Population Parameters, Expression Bias, and Gene Function for Loci on the Neo-X Chromosome (247 KB DOC)

Table S3 Major Functional Categories of Genes Located on the Ancestral X and the Neo-X Chromosome (57 KB DOC)

Table S4 Number of Genes Located on the Ancestral X and the Neo-X Chromosome with Male-Biased, Female-Biased, and Nonbiased Expression (KB DOC).

Table S5 Origin of Drosophila miranda Strains Used for Sequence Analysis (38 KB DOC)

Table S6 Two-Class HKA Test (43 KB DOC)

Table S7 Numbers of Loci Showing Evidence for Recent Adaptive Evolution (22 KB DOC)

## Abbreviations

CLR test: composite likelihood ratio test
GOF test: goodness-of-fit test
HKA test: Hudson-Kreitman-Aguadé test
LD: linkage disequilibrium
MAP: maximum a posteriori
MCLS test: maximized composite likelihood surface test
MY: million years

## References

[pbio-1000082-b001] Charlesworth B, Charlesworth D (2000). The degeneration of Y chromosomes. Philos Trans R Soc Lond B Biol Sci.

[pbio-1000082-b002] Bull JJ (1983). Evolution of sex determining mechanisms.

[pbio-1000082-b003] Skaletsky H, Kuroda-Kawaguchi T, Minx P, Cordum H, Hillier L (2003). The male-specific region of the human Y chromosome is a mosaic of discrete sequence classes. Nature.

[pbio-1000082-b004] Bachtrog D (2003). Adaptation shapes patterns of genome evolution in sexual and asexual genomes in Drosophila. Nat Genet.

[pbio-1000082-b005] Guttman D, Charlesworth D (1998). An X-linked gene with a degenerate Y-linked homologue in a dioecious plant. Nature.

[pbio-1000082-b006] Vicoso B, Charlesworth B (2006). Evolution on the X chromosome: unusual patterns and processes. Nat Rev Genet.

[pbio-1000082-b007] Ellegren H, Parsch J (2007). The evolution of sex-biased genes and sex-biased gene expression. Nat Rev Genet.

[pbio-1000082-b008] Singh ND, Petrov DA (2007). Evolution of gene function on the X chromosome versus the autosomes. Genome Dyn.

[pbio-1000082-b009] Gurbich TA, Bachtrog D (2008). Gene content evolution on the X chromosome. Curr Opin Genet Dev.

[pbio-1000082-b010] Marin I, Siegal ML, Baker BS (2000). The evolution of dosage-compensation mechanisms. Bioessays.

[pbio-1000082-b011] Bone JR, Kuroda MI (1996). Dosage compensation regulatory proteins and the evolution of sex chromosomes in Drosophila. Genetics.

[pbio-1000082-b012] Marin I, Franke A, Bashaw G, Baker B (1996). The dosage compensation system of Drosophila is co-opted by newly evolved X chromosomes. Nature.

[pbio-1000082-b013] Filatov DA (2008). A selective sweep in or near the Silene latifolia X-linked gene SlssX. Genet Res.

[pbio-1000082-b014] Charlesworth B, Coyne JA, Barton NH (1987). The relative rates of evolution of sex chromosomes and autosomes. Am Nat.

[pbio-1000082-b015] Rice WR (1984). Sex chromosomes and the evolution of sexual dimorphism. Evolution.

[pbio-1000082-b016] Khil P, Smirnova N, Romanienko P, Camerini-Otero R (2004). The mouse X chromosome is enriched for sex-biased genes not subject to selection by meiotic sex chromosome inactivation. Nat Genet.

[pbio-1000082-b017] Sturgill D, Zhang Y, Parisi M, Oliver B (2007). Demasculinization of X chromosomes in the Drosophila genus. Nature.

[pbio-1000082-b018] Evans AL, Mena PA, McAllister BF (2007). Positive selection near an inversion breakpoint on the neo-X chromosome of Drosophila americana. Genetics.

[pbio-1000082-b019] Lucchesi JC (1978). Gene dosage compensation and the evolution of sex chromosomes. Science.

[pbio-1000082-b020] Steinemann M, Steinemann S (1998). Enigma of Y chromosome degeneration: neo-Y and neo-X chromosomes of Drosophila miranda a model for sex chromosome evolution. Genetica.

[pbio-1000082-b021] Charlesworth B (1996). The evolution of chromosomal sex determination and dosage compensation. Curr Biol.

[pbio-1000082-b022] Tamura K, Subramanian S, Kumar S (2004). Temporal patterns of fruit fly (Drosophila) evolution revealed by mutation clocks. Mol Biol Evol.

[pbio-1000082-b023] Bachtrog D, Charlesworth B (2002). Reduced adaptation of a non-recombining neo-Y chromosome. Nature.

[pbio-1000082-b024] Bachtrog D, Hom E, Wong K, Maside X, de Jong P (2008). Genomic degradation of a young Y chromosome in Drosophila miranda. Genome Biol.

[pbio-1000082-b025] Wall J, Andolfatto P, Przeworski M (2002). Testing models of selection and demography in Drosophila simulans. Genetics.

[pbio-1000082-b026] Charlesworth B (2001). The effect of life-history and mode of inheritance on neutral genetic variability. Genet Res.

[pbio-1000082-b027] Begun D, Whitley P (2000). Reduced X-linked nucleotide polymorphism in Drosophila simulans. Proc Natl Acad Sci U S A.

[pbio-1000082-b028] Bachtrog D (2008). Similar rates of protein adaptation in Drosophila miranda and D. melanogaster, two species with different effective population sizes. BMC Evol Biol.

[pbio-1000082-b029] Maynard Smith J, Haigh J (1974). The hitch-hiking effect of a favourable gene. Genet Res.

[pbio-1000082-b030] Hudson RR, Kreitman M, Aguade M (1987). A test of neutral molecular evolution based on nucleotide data. Genetics.

[pbio-1000082-b031] Sackton TB, Lazzaro BP, Schlenke TA, Evans JD, Hultmark D (2007). Dynamic evolution of the innate immune system in Drosophila. Nat Genet.

[pbio-1000082-b032] Proschel M, Zhang Z, Parsch J (2006). Widespread adaptive evolution of Drosophila genes with sex-biased expression. Genetics.

[pbio-1000082-b033] Tajima F (1989). Statistical method for testing the neutral mutation hypothesis by DNA polymorphism. Genetics.

[pbio-1000082-b034] Jensen J, Kim Y, Dumont V, Aquadro C, Bustamante C (2005). Distinguishing between selective sweeps and demography using DNA polymorphism data. Genetics.

[pbio-1000082-b035] Braverman J, Hudson R, Kaplan N, Langley C, Stephan W (1995). The hitchhiking effect on the site frequency spectrum of DNA polymorphisms. Genetics.

[pbio-1000082-b036] Fay JC, Wu CI (2000). Hitchhiking under positive Darwinian selection. Genetics.

[pbio-1000082-b037] Fu YX (1997). Statistical tests of neutrality of mutations against population growth, hitchhiking and background selection. Genetics.

[pbio-1000082-b038] Kim Y, Stephan W (2002). Detecting a local signature of genetic hitchhiking along a recombining chromosome. Genetics.

[pbio-1000082-b039] Kim Y, Nielsen R (2004). Linkage disequilibrium as a signature of selective sweeps. Genetics.

[pbio-1000082-b040] Stephan W, Song Y, Langley C (2006). The hitchhiking effect on linkage disequilibrium between linked neutral loci. Genetics.

[pbio-1000082-b041] Jensen J, Thornton K, Bustamante C, Aquadro C (2007). On the utility of linkage disequilibrium as a statistic for identifying targets of positive selection in nonequilibrium populations. Genetics.

[pbio-1000082-b042] Yi S, Bachtrog D, Charlesworth B (2003). A survey of chromosomal and nucleotide sequence variation in Drosophila miranda. Genetics.

[pbio-1000082-b043] Bartolomé C, Charlesworth B (2006). Rates and patterns of chromosomal evolution in Drosophila pseudoobscura and D. miranda. Genetics.

[pbio-1000082-b044] Bachtrog D (2005). Sex chromosome evolution: molecular aspects of Y chromosome degeneration in Drosphila. Genome Res.

[pbio-1000082-b045] Nielsen R, Williamson S, Kim Y, Hubisz M, Clark A (2005). Genomic scans for selective sweeps using SNP data. Genome Res.

[pbio-1000082-b046] Wall JD (2000). A comparison of estimators of the population recombination rate. Mol Biol Evol.

[pbio-1000082-b047] Wiehe TH, Stephan W (1993). Analysis of a genetic hitchhiking model, and its application to DNA polymorphism data from Drosophila melanogaster. Mol Biol Evol.

[pbio-1000082-b048] Jensen J, Thornton K, Andolfatto P (2008). Quantifying the rate and strength of recurrent positive selection using DNA polymorphism data. PLoS Genetics.

[pbio-1000082-b049] Orr H (2005). The genetic theory of adaptation: a brief history. Nat Rev Genet.

[pbio-1000082-b050] Hudson R (2002). Generating samples under a Wright-Fisher neutral model of genetic variation. Bioinformatics.

[pbio-1000082-b051] Beaumont MA, Zhang W, Balding DJ (2002). Approximate Bayesian computation in population genetics. Genetics.

