# Supplementary figures and images for: Accelerated Adaptive Evolution on a Newly Formed X Chromosome
Source: PLoS Biol. 2009 Apr 14;7(4):e1000082. doi: 10.1371/journal.pbio.1000082 (PMC2672600; doi:10.1371/journal.pbio.1000082)

**X**

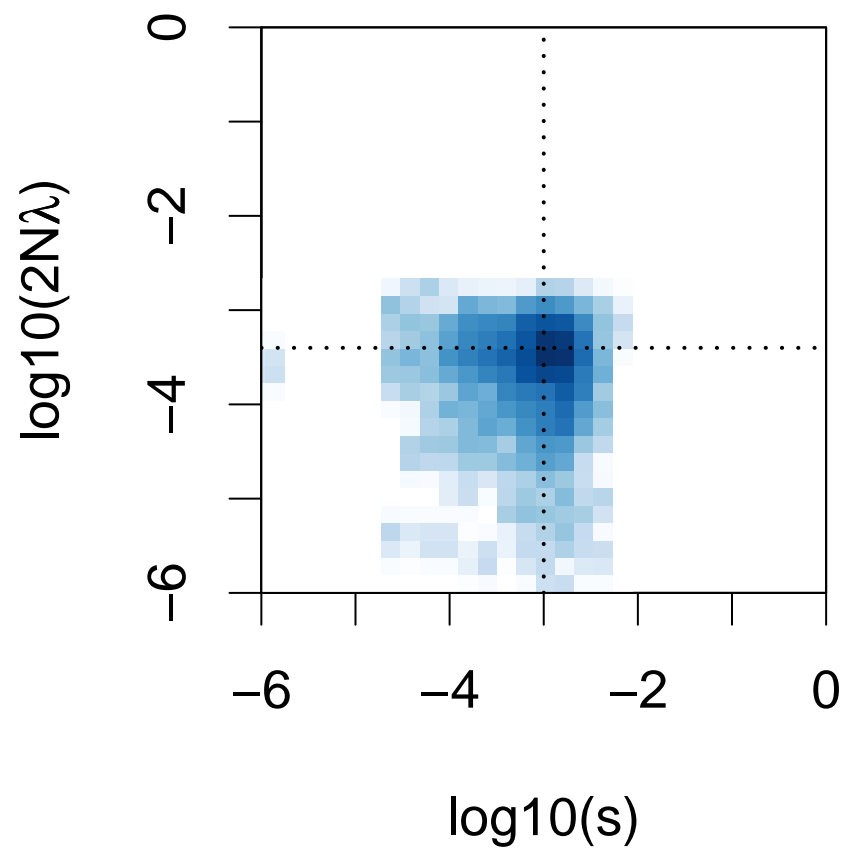

**neo-X**

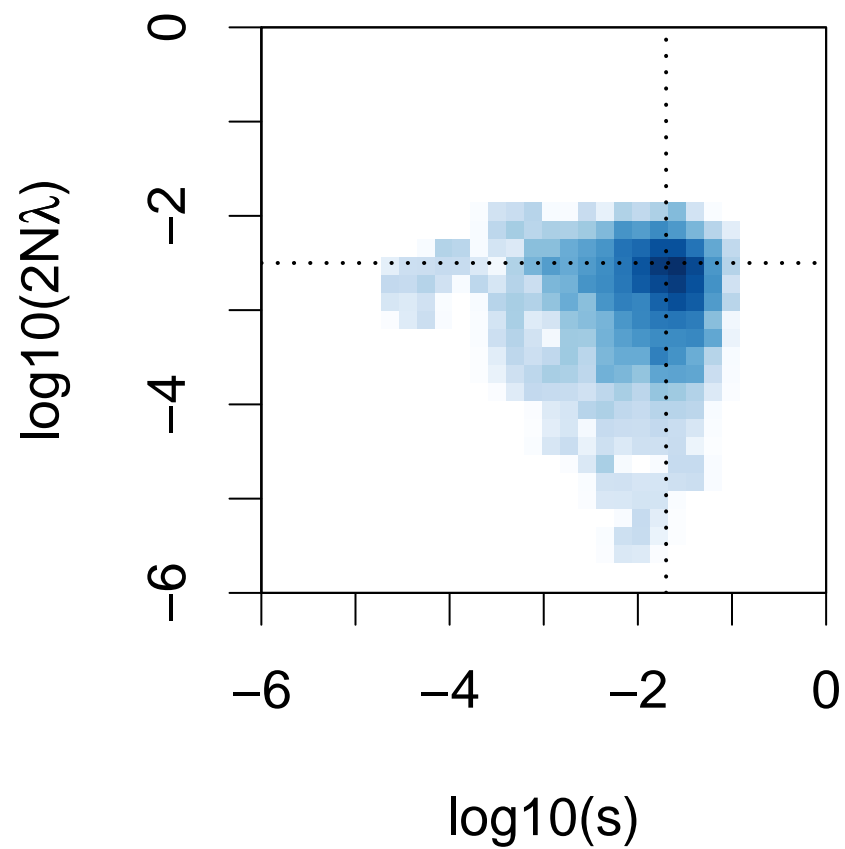

**strength of selection**

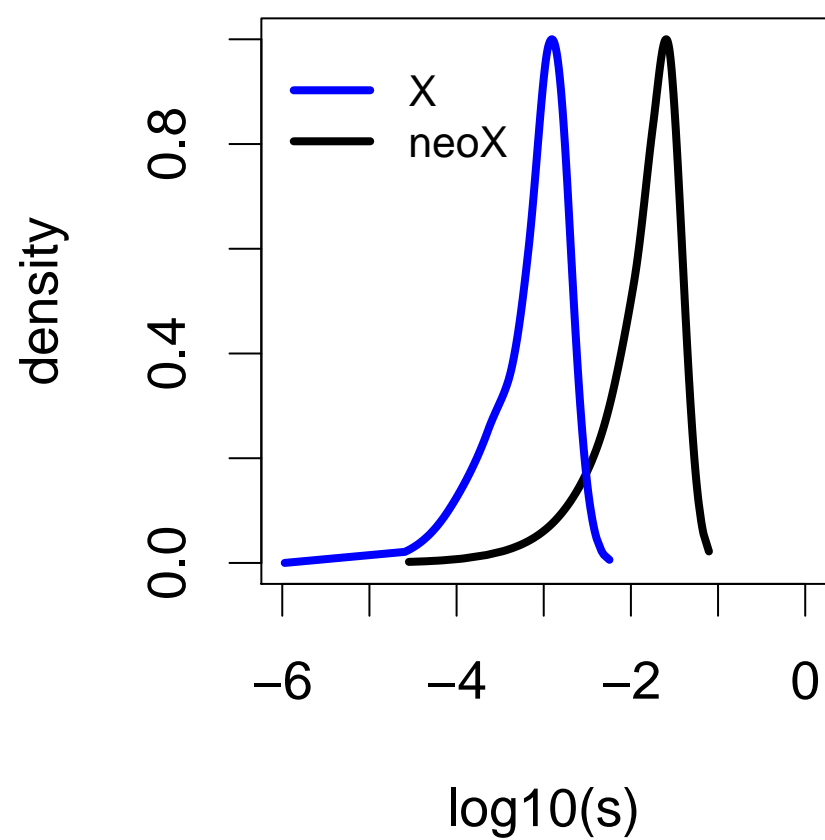

**rate of sweeps**

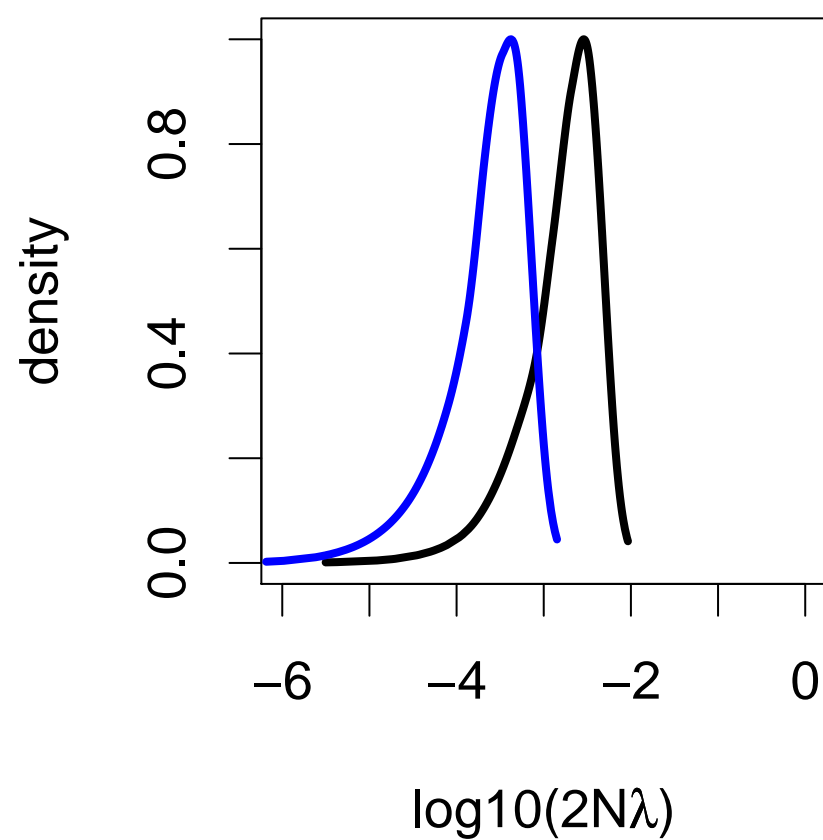

Supplement: Figure S1 — Approximate Bayesian estimation of the rate of adaptive substitutions (2Nλ) and their average effect on fitness (s) for genes on the ancestral X and the neo-X chromosome. Estimation is based on 106 draws from the prior (s ∼ Uniform (1.0E−06, 1.0) and 2Ne λ ∼ Uniform (1.0E−07, 1.0E−01)), where the selection parameters within a given replicate dataset are given by exponential distributions (see Materials and Methods for details on the inference procedure). (A) The joint posterior distributions for the X and neo-X. The dotted lines correspond to MAP estimates, and darker regions indicate greater posterior density. (B) The marginal posterior distribution for the X is indicated in blue, and for the neo-X in black. (105 KB PDF) [file pbio.1000082.sg001.pdf]
