# Supplementary material for: Accelerated Adaptive Evolution on a Newly Formed X Chromosome
Source: PLoS Biol. 2009 Apr 14;7(4):e1000082. doi: 10.1371/journal.pbio.1000082 (PMC2672600; doi:10.1371/journal.pbio.1000082)
Supplement: Table S1 — (188 KB DOC) [file pbio.1000082.st001.doc]

**Table S1. Locus-specific estimates of population parameters, expression bias and gene function for loci on the ancestral X chromosome.**

|  |  |  |  |  | Tests of selection | | |  | Expression Bias | | |  |  |
| --- | --- | --- | --- | --- | --- | --- | --- | --- | --- | --- | --- | --- | --- |
| Locus | Sample | Syn. | Seg. | π_syn_^f^ | CLRT ^g^ | GOF ^h^ | Ω ^i^ |  | Female/Male |  |  |  | GO categories ^l^ |
|  | Size | Sites ^d^ | Sites ^e^ |  | p-value | p-value | p-value |  | ratio ^j^ | p-value | bias ^k^ |  |  |
| GA23664 ^b^ | 17 | 1062 | 15 | 0.0079 | 0.2500 | NA | 0.1400 |  | — | — | — |  | — |
| GA28423 ^b^ | 17 | 1095 | 4 | 0.0034 | 0.6540 | NA | 0.1090 |  | -0.2035 | 0.3040 | N |  | 0003774,0003824,0005198,  0005488 |
| GA10077 ^b^ | 17 | 1098 | 1 | 0.0008 | 0.3560 | NA | 0.6550 |  | 0.5130 | 0.0070 | F |  | 0003824 |
| GA10095 ^b^ | 16 | 1170 | 16 | 0.0123 | 0.3540 | NA | 0.6420 |  | -0.1458 | 0.5672 | N |  | 0003824 |
| GA10179 ^b^ | 17 | 1089 | 9 | 0.0090 | 0.7650 | NA | 0.0980 |  | 0.1198 | 0.8578 | N |  | 0003824,0005198,0005488 |
| GA10984 ^b^ | 16 | 1068 | 7 | 0.0047 | 0.6350 | NA | 0.7540 |  | 0.7925 | 0.0000 | F |  | — |
| GA11427 ^b^ | 16 | 1080 | 6 | 0.0032 | 0.9870 | NA | 0.7650 |  | 0.2562 | 0.2668 | N |  | 0005488 |
| GA11430 ^b^ | 13 | 1104 | 19 | 0.0174 | 0.0660 | NA | 0.1220 |  | -0.0710 | 0.6497 | N |  | 0005488,0060089 |
| GA11437 ^b^ | 16 | 1062 | 22 | 0.0204 | 0.0980 | NA | 0.2130 |  | -0.8372 | 0.0000 | M |  | 0003824,0005488 |
| GA11471 ^b^ | 15 | 960 | 13 | 0.0185 | 0.2650 | NA | 0.5340 |  | 0.1602 | 0.6973 | N |  | 0003824,0016209 |
| GA11778 ^b^ | 17 | 1125 | 3 | 0.0035 | NA | NA | NA |  | 0.6026 | 0.0004 | F |  | — |
| GA23665 ^b^ | 17 | 1140 | 11 | 0.0072 | 1.0000 | NA | 0.2030 |  | -1.1563 | 0.0000 | M |  | — |
| GA12234 ^b^ | 17 | 1128 | 4 | 0.0013 | 0.6450 | NA | 0.2540 |  | 0.9506 | 0.0000 | F |  | 0005198 |
| GA20468 ^b^ | 16 | 1143 | 2 | 0.0009 | 0.2340 | NA | 0.6600 |  | 0.7455 | 0.0000 | F |  | 0005488,0060089 |
| GA12483 ^b^ | 17 | 1065 | 12 | 0.0086 | 0.5430 | NA | 0.2400 |  | 2.4675 | 0.0000 | F |  | 0005198,0005488,0060089 |
| GA12611 ^b^ | 17 | 1119 | 4 | 0.0034 | 0.7650 | NA | 0.6450 |  | 0.9225 | 0.0000 | F |  | 0005488 |
| GA12618 ^b^ | 17 | 1089 | 13 | 0.0104 | 0.2650 | NA | 0.7560 |  | -0.0796 | 0.4292 | N |  | — |
| GA12624 ^b^ | 17 | 660 | 7 | 0.0112 | 0.3540 | NA | 0.6540 |  | 0.0288 | 0.8546 | N |  | — |
| GA12731 ^b^ | 17 | 1122 | 5 | 0.0013 | 0.2340 | NA | 0.2340 |  | -0.4904 | 0.0001 | M |  | — |
| GA22263 ^b^ | 16 | 1053 | 0 | 0.0000 | NA | NA | NA |  | 0.1270 | 0.8964 | N |  | 0005488 |
| GA13389 ^b^ | 11 | 1137 | 6 | 0.0025 | 0.1340 | NA | 0.2330 |  | -0.1217 | 0.4534 | N |  | 0003824,0005198,0005488,  0060089 |
| GA13546 ^b^ | 17 | 1119 | 15 | 0.0135 | 0.1400 | NA | 0.7350 |  | -0.0129 | 0.7476 | N |  | 0005198,0005215 |
| GA13798 ^b^ | 17 | 1026 | 4 | 0.0048 | 0.6540 | NA | 0.2650 |  | 0.5925 | 0.0537 | N |  | 0003824,0005488 |
| GA14061 ^b^ | 16 | 1083 | 1 | 0.0005 | 1.0000 | NA | 0.3760 |  | 0.0829 | 0.7048 | N |  | 0005488 |
| GA14068 ^c^ | 12 | 618 | 9 | 0.0084 | 0.5600 | NA | 0.2540 |  | 0.0795 | 0.8964 | N |  | 0005488,0030528 |
| GA14078 ^b^ | 11 | 975 | 1 | 0.0000 | 0.2650 | NA | 0.7860 |  | 0.3909 | 0.0021 | F |  | 0005215,0030234 |
| GA14229 ^c^ | 12 | 711 | 1 | 0.0010 | 0.9650 | NA | 0.1100 |  | 0.3693 | 0.0000 | F |  | 0005215,0005488 |
| GA14350 ^b^ | 17 | 1104 | 5 | 0.0047 | 0.3760 | NA | 0.3540 |  | 0.1370 | 0.6306 | N |  | 0003824 |
| GA14357 ^b^ | 17 | 1073 | 5 | 0.0061 | 0.8750 | NA | 0.4870 |  | 0.5078 | 0.0011 | F |  | 0003824,0005488,0030528 |
| GA14442 ^b^ | 17 | 1086 | 3 | 0.0013 | 0.5650 | NA | 0.4230 |  | 0.6672 | 0.0017 | F |  | — |
| GA14613 ^b^ | 17 | 1131 | 9 | 0.0055 | 1.0000 | NA | 0.2540 |  | -0.6295 | 0.0006 | M |  | — |
| GA14619 ^b^ | 13 | 1122 | 6 | 0.0048 | 0.6540 | NA | 0.0890 |  | 0.1446 | 0.7802 | N |  | 0005488,0060089 |
| GA14649 ^b^ | 17 | 1086 | 5 | 0.0058 | 0.4760 | NA | 0.3560 |  | -0.0729 | 0.6941 | N |  | — |
| GA14862 ^b^ | 14 | 1113 | 13 | 0.0063 | 0.7690 | NA | 0.7600 |  | 0.0116 | 0.9093 | N |  | 0005488 |
| GA14956 ^c^ | 12 | 342 | 5 | 0.0127 | 0.2650 | NA | 0.6540 |  | 0.0543 | 0.9412 | N |  | 0005488,0030528 |
| GA15129 ^b^ | 17 | 1149 | 18 | 0.0206 | 0.1320 | NA | 0.5400 |  | -0.2948 | 0.0998 | N |  | 0003824,0005198 |
| GA15293 ^b^ | 17 | 1104 | 20 | 0.0185 | 0.2110 | NA | 0.6520 |  | -0.3081 | 0.2452 | N |  | 0003824,0005488 |
| GA15324 ^b^ | 13 | 984 | 3 | 0.0024 | 0.6340 | NA | 0.2720 |  | 0.3436 | 0.0655 | N |  | — |
| GA15328 ^b^ | 13 | 1080 | 10 | 0.0096 | 0.8760 | NA | 0.2520 |  | 0.1734 | 0.7542 | N |  | — |
| GA15408 ^c^ | 12 | 651 | 3 | 0.0030 | 0.2430 | NA | 0.5340 |  | -0.0168 | 0.8480 | N |  | 0003824,0005488,0030528,  0060089 |
| GA15408 ^c^ | 12 | 1155 | 14 | 0.0057 | 0.7450 | NA | 0.9570 |  | -0.0168 | 0.8480 | N |  | 0003824,0005488,0030528,  0060089 |
| GA15557 ^b^ | 16 | 1008 | 10 | 0.0026 | 0.6530 | NA | 0.7760 |  | -0.9130 | 0.0000 | M |  | 0003824 |
| GA15911 ^b^ | 16 | 1083 | 2 | 0.0000 | 0.2540 | NA | 0.7680 |  | -0.2533 | 0.0364 | N |  | 0060089 |
| GA15921 ^b^ | 16 | 1119 | 2 | 0.0019 | 0.2640 | NA | 0.8760 |  | -0.0340 | 0.8718 | N |  | 0005488 |
| GA16706 ^b^ | 14 | 1032 | 5 | 0.0062 | **0.008*** | **0.553*** | **0.017*** |  | -0.8372 | 0.0000 | M |  | 0005198,0005488 |
| GA16760 ^b^ | 17 | 1095 | 5 | 0.0060 | 0.8000 | NA | 0.4340 |  | 0.2213 | 0.4210 | N |  | 0005488 |
| GA16811 ^b^ | 17 | 1110 | 4 | 0.0039 | 0.8530 | NA | 0.4400 |  | 0.2426 | 0.1838 | N |  | 0005488,0030528 |
| GA16824 ^b^ | 17 | 1128 | 22 | 0.0243 | 0.2540 | NA | 0.5980 |  | -0.1143 | 0.5267 | N |  | 0005198 |
| GA16990 ^b^ | 17 | 1047 | 17 | 0.0217 | 0.3500 | NA | 0.2540 |  | 0.3229 | 0.0151 | N |  | 0005488,0030234 |
| GA16991 ^b^ | 16 | 1146 | 2 | 0.0016 | 0.7560 | NA | 0.3540 |  | 0.1283 | 0.6447 | N |  | 0005488 |
| GA17331 ^b^ | 17 | 1140 | 15 | 0.0065 | 0.3460 | NA | 0.5240 |  | 0.2404 | 0.1175 | N |  | 0005488,0045182 |
| GA17446 ^c^ | 12 | 969 | 3 | 0.0018 | 0.4650 | NA | 0.2650 |  | 1.3655 | 0.0000 | F |  | 0005488 |
| GA17564 ^b^ | 16 | 1047 | 3 | 0.0016 | 0.2340 | NA | 0.2540 |  | -0.0478 | 0.8454 | N |  | 0003824,0005488,0030234 |
| GA17698 ^b^ | 17 | 1098 | 4 | 0.0025 | 0.6520 | NA | 0.1320 |  | 0.4806 | 0.0000 | F |  | 0005488,0030234,0045182 |
| GA18065 ^c^ | 12 | 543 | 0 | 0.0000 | NA | NA | NA |  | 0.0151 | 0.7399 | N |  | 0005488 |
| GA18255 ^b^ | 17 | 1092 | 4 | 0.0005 | 0.8740 | NA | 0.1100 |  | 0.2325 | 0.4983 | N |  | 0005488,0030528 |
| GA18482 ^b^ | 16 | 1080 | 3 | 0.0005 | 0.5240 | NA | 0.1540 |  | 0.6990 | 0.0000 | F |  | — |
| GA18581 ^b^ | 15 | 1083 | 8 | 0.0043 | 0.2240 | NA | 0.6540 |  | -0.0688 | 0.7727 | N |  | 0003824 |
| GA19171 ^b^ | 17 | 1107 | 7 | 0.0052 | 0.2540 | NA | 0.3550 |  | 0.1563 | 0.5860 | N |  | 0003824,0005488,0060089 |
| GA19599 ^b^ | 17 | 1161 | 12 | 0.0064 | 0.7560 | NA | 0.5650 |  | 0.0265 | 0.9178 | N |  | 0005488 |
| GA19604 ^b^ | 15 | 996 | 1 | 0.0000 | 0.2650 | NA | 0.3760 |  | 0.1538 | 0.5850 | N |  | — |
| GA19855 ^b^ | 15 | 735 | 10 | 0.0043 | 0.8760 | NA | 0.3760 |  | -0.1921 | 0.1953 | N |  | 0005488 |
| GA19954 ^b^ | 17 | 1074 | 11 | 0.0133 | 0.9860 | NA | 0.8760 |  | -0.0476 | 0.6403 | N |  | 0005215,0005488,0030234 |
| GA20037 ^b^ | 17 | 1119 | 7 | 0.0058 | 0.6250 | NA | 0.2430 |  | 0.2838 | 0.0370 | N |  | 0003824,0005488 |
| GA20049 ^b^ | 16 | 990 | 3 | 0.0029 | 0.2430 | NA | 0.6520 |  | 0.4064 | 0.0021 | F |  | 0005488,0030528 |
| GA20060 ^b^ | 17 | 1173 | 10 | 0.0091 | 0.1230 | NA | 0.2650 |  | -1.1615 | 0.0000 | M |  | 0003774,0003824,0005198 |
| GA20094 ^b^ | 15 | 1110 | 8 | 0.0072 | 0.0860 | NA | 0.6540 |  | -0.2035 | 0.3040 | N |  | 0003774,0003824,0005198,  0005488 |
| GA20157 ^b^ | 17 | 1164 | 5 | 0.0031 | 0.2340 | NA | 0.6760 |  | 0.2304 | 0.1041 | N |  | 0003824,0005488,0060089 |
| GA20274 ^b^ | 17 | 1128 | 4 | 0.0057 | 0.5600 | NA | 0.8760 |  | 0.8052 | 0.0000 | F |  | — |
| GA20355 ^b^ | 16 | 1041 | 3 | 0.0005 | 1.0000 | NA | 0.9850 |  | -1.5885 | 0.0000 | M |  | 0003824,0005488 |
| GA20381 ^b^ | 17 | 990 | 5 | 0.0018 | 0.6540 | NA | 0.4620 |  | -0.2055 | 0.0779 | N |  | 0003824,0005488 |
| GA20400 ^b^ | 13 | 981 | 11 | 0.0132 | **0.023*** | 0.0210 | 0.2430 |  | -0.2035 | 0.3040 | N |  | 0003774,0003824,0005198,  0005488 |
| GA10464 ^b^ | 17 | 942 | 18 | 0.0174 | 0.6980 | NA | 0.2650 |  | 0.5311 | 0.0006 | F |  | 0003824,0005488,0060089 |
| GA20622 ^b^ | 16 | 1083 | 12 | 0.0053 | 0.2650 | NA | 0.7650 |  | 0.7791 | 0.0000 | F |  | 0005488,0030528 |
| GA20654 ^b^ | 17 | 1020 | 3 | 0.0030 | 0.3540 | NA | 0.4560 |  | 0.2347 | 0.2403 | N |  | 0005488 |
| GA20797 ^b^ | 16 | 1068 | 11 | 0.0094 | 0.1320 | NA | 0.5430 |  | 0.0878 | 0.9591 | N |  | 0005215 |
| GA20870 ^b^ | 17 | 1149 | 16 | 0.0155 | 0.1430 | NA | 0.2400 |  | 0.2432 | 0.0012 | F |  | 0005215 |
| GA21020 ^b^ | 9 | 990 | 9 | 0.0100 | 0.1540 | NA | 0.3540 |  | 0.1374 | 0.0558 | N |  | 0003824,0005488 |
| GA21098 ^b^ | 17 | 1155 | 4 | 0.0017 | 0.5430 | NA | 0.2540 |  | 0.1511 | 0.2179 | N |  | — |
| GA21186 ^b^ | 16 | 1161 | 7 | 0.0043 | **0.021*** | 0.0430 | 0.2640 |  | 0.4376 | 0.0242 | N |  | 0003774,0005198,0005488 |
| GA21397 ^c^ | 12 | 768 | 0 | 0.0000 | NA | NA | NA |  | 0.3258 | 0.0364 | N |  | 0003824,0005488 |
| GA21472 ^b^ | 16 | 1095 | 6 | 0.0041 | 0.2540 | NA | 0.7600 |  | 0.4152 | 0.0343 | N |  | 0005488 |
| GA21606 ^b^ | 16 | 1140 | 6 | 0.0061 | 0.2500 | NA | 0.8340 |  | 0.0881 | 0.5157 | N |  | 0003824 |
| GA21664 ^b^ | 16 | 1083 | 13 | 0.0095 | 0.3650 | NA | 0.6030 |  | -1.2081 | 0.0000 | M |  | 0003774,0005198 |
| GA21956 ^b^ | 17 | 1002 | 2 | 0.0009 | 0.7560 | NA | 0.2560 |  | 0.0219 | 0.8297 | N |  | 0003824,0005488 |
| GA22026 ^b^ | 17 | 1116 | 3 | 0.0014 | 0.8750 | NA | 0.6450 |  | -0.0784 | 0.2995 | N |  | 0003824,0005488,0060089 |
| GA22111 ^b^ | 15 | 1083 | 4 | 0.0011 | 1.0000 | NA | 0.7000 |  | 0.0374 | 0.9267 | N |  | 0005198 |
| GA22200 ^b^ | 13 | 1071 | 19 | 0.0118 | 0.8800 | NA | 0.8760 |  | -0.0338 | 0.6666 | N |  | 0003824 |
| GA22350 ^b^ | 16 | 1047 | 11 | 0.0036 | 0.4320 | NA | 0.6540 |  | 0.1217 | 0.7223 | N |  | — |
| GA22600 ^b^ | 15 | 1167 | 4 | 0.0000 | 0.2530 | NA | 0.4330 |  | -1.6315 | 0.0000 | M |  | 0003824 |
| GA15192 ^b^ | 17 | 1149 | 12 | 0.0115 | 0.5340 | NA | 0.2540 |  | -1.1683 | 0.0000 | M |  | 0003824 |
| GA22667 ^b^ | 16 | 1002 | 6 | 0.0036 | 0.2400 | NA | 0.2540 |  | -0.1363 | 0.4383 | N |  | — |
| GA22806 ^c^ | 12 | 612 | 1 | 0.0000 | 0.1430 | NA | 0.8760 |  | 0.1465 | 0.2538 | N |  | 0005488 |
| GA16961 ^b^ | 16 | 1104 | 3 | 0.0005 | 0.3540 | NA | 0.5240 |  | -0.0285 | 0.7878 | N |  | 0003824,0005488,0045182 |
| GA22854 ^b^ | 16 | 1089 | 1 | 0.0000 | 1.0000 | NA | 0.5430 |  | 0.0202 | 0.9894 | N |  | — |
| GA22930 ^b^ | 15 | 1068 | 11 | 0.0094 | 0.1540 | NA | 0.5400 |  | -0.4326 | 0.0008 | M |  | 0005488,0030234 |
| GA23024 ^b^ | 16 | 1092 | 9 | 0.0062 | 0.3570 | NA | 0.6250 |  | 0.1721 | 0.2538 | N |  | 0005488 |
| GA23405 ^b^ | 17 | 1143 | 5 | 0.0053 | 0.1500 | NA | 0.7650 |  | 0.4175 | 0.0282 | N |  | 0003824,0005488 |
| GA23503 ^b^ | 14 | 600 | 10 | 0.0234 | 0.1530 | NA | 0.8360 |  | 0.2818 | 0.1216 | N |  | 0003824,0005488,0060089 |
| GA23532 ^b^ | 16 | 1161 | 4 | 0.0020 | 0.1100 | NA | 0.3760 |  | — | — | — |  | 0005488 |
| GA23653 ^b^ | 17 | 1089 | 7 | 0.0113 | 0.2430 | NA | 0.2650 |  | 0.1337 | 0.7835 | N |  | 0003824 |
| GA23736 ^b^ | 17 | 1107 | 5 | 0.0048 | 0.2600 | NA | 0.2110 |  | -0.1271 | 0.5559 | N |  | — |
| GA24110 ^b^ | 16 | 1068 | 16 | 0.0072 | 0.2660 | NA | 0.1230 |  | 1.2055 | 0.0000 | F |  | 0005198 |
| GA25899 ^a^ | 12 | 810 | 3 | 0.0008 | 0.4560 | NA | 0.2650 |  | 0.3463 | 0.0676 | N |  | — |
| GA27356 ^b^ | 17 | 1101 | 2 | 0.0000 | 0.6500 | NA | 0.3050 |  | 0.4513 | 0.0015 | F |  | 0005488,0045182 |
| GA27380 ^b^ | 16 | 1041 | 2 | 0.0013 | 0.2500 | NA | 0.3650 |  | 0.5205 | 0.0000 | F |  | 0003774,0005488 |
| GA27422 ^b^ | 16 | 60 | 1 | 0.0082 | 0.6350 | NA | 0.8090 |  | -0.1865 | 0.0099 | M |  | 0003824 |
| GA28121 ^b^ | 16 | 1089 | 1 | 0.0000 | 0.4500 | NA | 0.3300 |  | -0.9077 | 0.0000 | M |  | 0005215,0060089 |
| GA28220 ^b^ | 17 | 1077 | 12 | 0.0140 | 0.1950 | NA | 0.7560 |  | -0.2226 | 0.2347 | N |  | 0003824,0005488,0060089 |
| GA28415 ^b^ | 16 | 993 | 7 | 0.0047 | 0.2650 | NA | 0.2340 |  | -0.2395 | 0.4034 | N |  | 0003824 |
| GA28541 ^b^ | 15 | 957 | 9 | 0.0066 | 0.9840 | NA | 0.2300 |  | -0.2035 | 0.3040 | N |  | 0003774,0003824,0005198,  0005488 |
| GA29095 ^c^ | 12 | 666 | 4 | 0.0037 | 0.7650 | NA | 0.2330 |  | 0.0201 | 0.9200 | N |  | 0005488,0030528 |

^a^ Bachtrog & Andolfatto 2006

^b^ this study & Bachtrog (2008) – Note: Bachtrog (2008) only studied 14 alleles.

^c^ Bartolome et al. 2006

^d^ Number of synonymous sites studies

^e^ Count of segregating synonymous sites

^f^ The weighted average within-species pairwise synonymous diversity per synonymous site.

^g^ Locus-specific p-value for the CLRT test (Kim & Stephan 2002)

^h^ Locus-specific p-value for the GOF test (Jensen et al. 2005)

^i^ Locus-specific p-value for the CLRT test (Kim & Nielsen 2004)

^j^ Locus-specific female/male expression ratios inferred from *D. pseudoobscura* (Sturgill et al. 2007).

^k^ Locus-specific expression bias (M: male-biased expression; F: female-biased expression, N: non-biased expression.

^l^ Locus-specific gene ontology classes extracted from FlyBase (<http://flybase.bio.indiana.edu/>)

Bachtrog, D and Andolfatto, P, Selection, recombination and demographic history in *Drosophila miranda*. *Genetics* **174** (4), 2045 (2006).

Bachtrog, D Similar rates of protein adaptation in *Drosophila miranda* and *D. melanogaster*, two species with different effective population sizes. *BMC Evolutionary Biology* **8,** 334 (2008).

Bartolomé, C et al., Patterns of selection on synonymous and nonsynonymous variants in Drosophila miranda. *Genetics* **169** (3), 1495-507 (2005).

Jensen, JD et al., Distinguishing between selective sweeps and demography using DNA polymorphism data. *Genetics* **170** (3), 1401 (2005).

Kim, Y and Stephan, W, Detecting a local signature of genetic hitchhiking along a recombining chromosome. *Genetics* **160** (2), 765 (2002).

Kim, Y and Nielsen, R, Linkage disequilibrium as a signature of selective sweeps. *Genetics* **167** (3), 1513 (2004).

Sturgill D, Zhang Y, Parisi M, Oliver B Demasculinization of X chromosome genes in the Drosophila genus. (2007). Nature **450**:238-241.
