# Supplementary material for: Accelerated Adaptive Evolution on a Newly Formed X Chromosome
Source: PLoS Biol. 2009 Apr 14;7(4):e1000082. doi: 10.1371/journal.pbio.1000082 (PMC2672600; doi:10.1371/journal.pbio.1000082)
Supplement: Table S2 — (247 KB DOC) [file pbio.1000082.st002.doc]

**Table S2. Locus-specific estimates of population parameters, expression bias and gene function for loci on the neo-X chromosome.**

|  |  |  |  |  | Tests of selection | | |  | Expression Bias | | |  |  |
| --- | --- | --- | --- | --- | --- | --- | --- | --- | --- | --- | --- | --- | --- |
| Locus | Sample | Syn. | Seg. | π_syn_^f^ | CLRT ^g^ | GOF ^h^ | Ω ^i^ |  | Female/Male |  |  |  | GO categories ^l^ |
|  | Size | Sites ^d^ | Sites ^e^ |  | p-value | p-value | p-value |  | ratio ^j^ | p-value | bias ^k^ |  |  |
| GA10102 ^a^ | 12 | 987 | 0 | 0.0000 | NA | NA | NA |  | -0.9910 | 0.0000 | M |  | 0003824 |
| GA10235 ^b^ | 16 | 1017 | 7 | 0.0064 | 0.355 | NA | 0.264 |  | -1.6699 | 0.0000 | M |  | 0003824,0005198,0060089 |
| GA10241 ^b^ | 14 | 948 | 0 | 0.0000 | NA | NA | NA |  | 0.3410 | 0.0015 | F |  | 0005488,0030528 |
| GA10242 ^b^ | 16 | 315 | 1 | 0.0016 | 0.383 | NA | 0.754 |  | -0.1089 | 0.5956 | N |  | 0003824 |
| GA10776 ^b^ | 17 | 1032 | 8 | 0.0035 | 0.392 | NA | 0.265 |  | 0.4334 | 0.0100 | N |  | — |
| GA10784 ^c^ | 18 | 1419 | 10 | 0.0082 | **0.038*** | **0.701*** | **0.013*** |  | -0.2346 | 0.1821 | N |  | — |
| GA10787 ^b^ | 17 | 891 | 2 | 0.0016 | 0.392 | NA | 0.763 |  | -1.1371 | 0.0000 | M |  | — |
| GA10804 ^c^ | 18 | 312 | 1 | 0.0016 | 0.053 | NA | 0.098 |  | -0.0901 | 0.8804 | N |  | 0003824 |
| GA10852 ^b^ | 17 | 1005 | 2 | 0.0022 | 0.397 | NA | 0.833 |  | 0.3493 | 0.0219 | N |  | 0003824,0005488,0060089 |
| GA10968 ^b^ | 15 | 564 | 4 | 0.0010 | 0.403 | NA | 0.11 |  | -0.1827 | 0.0490 | N |  | — |
| GA10970 ^b^ | 17 | 378 | 2 | 0.0014 | 0.407 | NA | 0.076 |  | -0.2044 | 0.0035 | M |  | 0005215 |
| GA11141^a^ | 12 | 1080 | 5 | 0.0033 | **0.001*** | **0.928*** | 0.238 |  | -0.0077 | 0.7856 | N |  | 0003824,0005488 |
| GA11265 ^c^ | 18 | 1647 | 34 | 0.0188 | **0.011*** | **0.562*** | **0.001*** |  | -0.2732 | 0.1677 | N |  | 0005488,0060089 |
| GA11400 ^b^ | 17 | 408 | 4 | 0.0044 | 0.423 | NA | 0.524 |  | 0.8165 | 0.0000 | F |  | — |
| GA11779 ^b^ | 17 | 1173 | 3 | 0.0036 | 0.455 | NA | 0.734 |  | 0.2768 | 0.0976 | N |  | 0003824,0005488,0045182 |
| GA11837 ^b^ | 17 | 387 | 5 | 0.0105 | 0.468 | NA | 0.545 |  | -0.1534 | 0.4260 | N |  | — |
| GA11912 ^b^ | 17 | 717 | 5 | 0.0007 | 0.532 | NA | 0.234 |  | 0.4169 | 0.0067 | F |  | — |
| GA12097 ^b^ | 15 | 1377 | 5 | 0.0025 | 0.577 | NA | 0.96 |  | -1.1794 | 0.0000 | M |  | — |
| GA14754 ^b^ | 15 | 1299 | 1 | 0.0011 | 0.599 | NA | 0.543 |  | 0.3045 | 0.0079 | F |  | 0003824,0005488 |
| GA12287 ^c^ | 18 | 363 | 2 | 0.0048 | **0.01*** | 0.032 | 0.65 |  | 0.0798 | 0.8177 | N |  | — |
| GA12341^a^ | 12 | 1119 | 16 | 0.0203 | 1 | NA | 0.243 |  | 0.0969 | 0.9520 | N |  | 0060089 |
| GA12347 ^b^ | 17 | 516 | 1 | 0.0000 | 0.603 | NA | 0.732 |  | -0.0672 | 0.7103 | N |  | — |
| GA12348 ^b^ | 17 | 582 | 3 | 0.0035 | 0.623 | NA | 0.543 |  | -0.0219 | 0.9399 | N |  | — |
| GA12350 ^b^ | 14 | 303 | 2 | 0.0000 | 0.666 | NA | 0.345 |  | — | — | — |  | — |
| GA12367 ^b^ | 17 | 681 | 2 | 0.0029 | 0.687 | NA | 0.764 |  | 0.0491 | 0.8314 | N |  | — |
| GA12370 ^a^ | 12 | 1071 | 7 | 0.0047 | **0.037*** | **0.654*** | 0.054 |  | -0.1478 | 0.3974 | N |  | 0005488,0060089 |
| GA12502 ^b^ | 17 | 966 | 7 | 0.0099 | 0.697 | NA | 0.234 |  | -0.1201 | 0.4201 | N |  | 0003824 |
| GA12504 ^b^ | 17 | 447 | 0 | 0.0000 | NA | NA | NA |  | -2.1012 | 0.0000 | M |  | — |
| GA12538 ^b^ | 17 | 1266 | 8 | 0.0046 | 0.732 | NA | 0.645 |  | -2.2527 | 0.0000 | M |  | 0005215 |
| GA12545 ^b^ | 17 | 663 | 1 | 0.0008 | 0.764 | NA | 0.777 |  | 1.5684 | 0.0000 | F |  | 0005198,0005488 |
| GA12583 ^a^ | 12 | 756 | 3 | 0.0043 | 0.053 | NA | 0.832 |  | 0.3268 | 0.0086 | F |  | 0030528 |
| GA12730 ^b^ | 17 | 792 | 1 | 0.0000 | **0.009*** | 0.05 | 0.342 |  | 0.4480 | 0.0077 | F |  | 0005488 |
| GA13871^b^ | 16 | 1050 | 9 | 0.0065 | 0.765 | NA | 0.23 |  | -0.1226 | 0.5482 | N |  | — |
| GA13876 ^a^ | 12 | 825 | 1 | 0.0008 | **0.041*** | 0.021 | 0.062 |  | 0.0370 | 0.9515 | N |  | — |
| GA13889 ^b^ | 15 | 597 | 0 | 0.0000 | NA | NA | NA |  | 0.2919 | 0.0317 | N |  | — |
| GA13944 ^b^ | 17 | 378 | 1 | 0.0013 | 0.832 | NA | 0.365 |  | 0.4903 | 0.0000 | F |  | 0005198 |
| GA14039 ^b^ | 17 | 654 | 0 | 0.0000 | NA | NA | NA |  | 0.6985 | 0.0000 | F |  | 0005215 |
| GA14043 ^b^ | 17 | 1164 | 1 | 0.0000 | 0.833 | NA | 0.835 |  | 0.3436 | 0.1300 | N |  | 0005488,0030234,0030528 |
| GA14047 ^b^ | 15 | 1203 | 1 | 0.0005 | 0.854 | NA | 0.354 |  | 0.6719 | 0.0000 | F |  | 0003824,0005488 |
| GA14051 ^b^ | 15 | 984 | 1 | 0.0006 | 0.899 | NA | 0.786 |  | 0.4136 | 0.0180 | N |  | 0005488 |
| GA14154 ^b^ | 17 | 264 | 1 | 0.0020 | 0.903 | NA | 0.76 |  | 1.1556 | 0.0000 | F |  | 0005488 |
| GA14314 ^c^ | 18 | 894 | 2 | 0.0017 | 0.245 | NA | 0.976 |  | 0.1320 | 0.5872 | N |  | 0003774,0005198,0005488 |
| GA14437 ^b^ | 17 | 327 | 3 | 0.0031 | 0.927 | NA | 0.987 |  | -0.4736 | 0.0002 | M |  | 0003824,0005215 |
| GA14533 ^b^ | 16 | 315 | 1 | 0.0029 | 0.927 | NA | 0.24 |  | 0.5735 | 0.0000 | F |  | — |
| GA14681 ^c^ | 18 | 990 | 6 | 0.0023 | 1 | NA | 0.623 |  | 0.3761 | 0.0201 | N |  | 0003824 |
| GA14754 ^b^ | 15 | 240 | 0 | 0.0000 | NA | NA | NA |  | 0.3045 | 0.0079 | F |  | — |
| GA14829 ^c^ | 18 | 762 | 0 | 0.0000 | NA | NA | NA |  | -0.1490 | 0.3855 | N |  | 0003824 |
| GA15112 ^c^ | 18 | 1368 | 5 | 0.0033 | 1 | NA | 0.876 |  | 0.1294 | 0.5278 | N |  | 0003824,0005488,0060089 |
| GA24647 ^a^ | 12 | 336 | 1 | 0.0050 | 1 | NA | 0.065 |  | 0.1647 | 0.5822 | N |  | 0005198 |
| GA15201 ^a^ | 12 | 336 | 3 | 0.0095 | **0.03*** | **0.882*** | 0.674 |  | 0.1647 | 0.5822 | N |  | 0005198 |
| GA15214 ^a^ | 12 | 1086 | 8 | 0.0026 | **0.007*** | **0.874*** | **0.017*** |  | -0.3069 | 0.0202 | N |  | 0003824,0005488 |
| GA15267 ^b^ | 14 | 339 | 1 | 0.0049 | 0.934 | NA | 0.264 |  | -0.3664 | 0.0000 | M |  | 0003774,0003824,0005198,  0005488 |
| GA15336 ^b^ | 17 | 1002 | 8 | 0.0016 | 0.956 | NA | 0.077 |  | -0.3309 | 0.0003 | M |  | — |
| GA15338 ^b^ | 17 | 690 | 4 | 0.0065 | 0.965 | NA | 0.065 |  | 0.2628 | 0.0523 | N |  | 0005488,0030528 |
| GA15349 ^a^ | 12 | 633 | 10 | 0.0152 | 0.299 | NA | 0.234 |  | -0.0383 | 0.8826 | N |  | 0003824,0005215,0005488,  0030528 |
| GA15578 ^b^ | 13 | 339 | 0 | 0.0000 | NA | NA | NA |  | -0.1511 | 0.1781 | N |  | 0005215,0005488 |
| GA15579 ^b^ | 17 | 1098 | 3 | 0.0013 | 0.987 | NA | 0.354 |  | -0.2463 | 0.0444 | N |  | 0003824,0005488 |
| GA15593 ^a^ | 12 | 999 | 11 | 0.0103 | 0.079 | NA | 0.978 |  | -0.1221 | 0.1464 | N |  | 0005215 |
| GA14650 ^b^ | 15 | 390 | 1 | 0.0029 | 0.998 | NA | 0.635 |  | -0.0274 | 0.7973 | N |  | 0003824,0005488,0060089 |
| GA15681 ^c^ | 18 | 474 | 0 | 0.0000 | NA | NA | NA |  | -0.0644 | 0.6820 | N |  | — |
| GA15697 ^b^ | 17 | 333 | 1 | 0.0031 | 1 | NA | 0.154 |  | -0.1517 | 0.1803 | N |  | — |
| GA15698 ^b^ | 14 | 105 | 0 | 0.0000 | 1 | NA | 0.101 |  | 1.0425 | 0.0000 | F |  | — |
| GA15709 ^b^ | 15 | 519 | 2 | 0.0000 | 1 | NA | 0.103 |  | 0.4335 | 0.0174 | N |  | — |
| GA24114 ^b^ | 14 | 324 | 2 | 0.0000 | 1 | NA | 0.323 |  | — | — | — |  | — |
| GA15731 ^a^ | 12 | 978 | 20 | 0.0197 | 0.099 | NA | 0.254 |  | 0.0553 | 0.9673 | N |  | — |
| GA15751 ^b^ | 15 | 192 | 2 | 0.0103 | 1 | NA | 0.635 |  | -0.2774 | 0.0549 | N |  | — |
| GA15755 ^b^ | 17 | 1002 | 2 | 0.0014 | 1 | NA | 0.765 |  | -0.1248 | 0.2974 | N |  | — |
| GA15879 ^b^ | 17 | 441 | 5 | 0.0118 | 1 | NA | 0.244 |  | -0.1122 | 0.2667 | N |  | 0005488 |
| GA15882 ^b^ | 12 | 888 | 4 | 0.0028 | 1 | NA | 0.545 |  | -0.1467 | 0.1289 | N |  | 0003824 |
| GA16095 ^b^ | 16 | 951 | 6 | 0.0022 | 1 | NA | 0.087 |  | -1.5419 | 0.0000 | M |  | 0005198,0005488 |
| GA16346 ^b^ | 17 | 1068 | 4 | 0.0049 | 1 | NA | 0.354 |  | 0.2528 | 0.0676 | N |  | — |
| GA16380 ^b^ | 17 | 1026 | 1 | 0.0005 | 1 | NA | 0.65 |  | 0.3360 | 0.0159 | N |  | — |
| GA16427 ^b^ | 17 | 1311 | 3 | 0.0019 | 1 | NA | 0.874 |  | 0.3504 | 0.0232 | N |  | — |
| GA16724 ^b^ | 17 | 1053 | 2 | 0.0014 | 1 | NA | 0.543 |  | -0.1262 | 0.4518 | N |  | 0003824,0005488,0060089 |
| GA16759 ^b^ | 17 | 1182 | 4 | 0.0025 | **0.021*** | **0.911*** | **0.012*** |  | 0.5718 | 0.0001 | F |  | — |
| GA17211 ^b^ | 16 | 1299 | 5 | 0.0036 | 1 | NA | 0.365 |  | 0.3088 | 0.0240 | N |  | — |
| GA17308 ^b^ | 16 | 1359 | 4 | 0.0027 | 1 | NA | 0.365 |  | 0.4795 | 0.0000 | F |  | 0005488,0030528 |
| GA17319 ^b^ | 16 | 1107 | 3 | 0.0026 | 1 | NA | 0.434 |  | 0.4400 | 0.0475 | N |  | 0003824 |
| GA17325 ^b^ | 17 | 1089 | 6 | 0.0026 | 1 | NA | 0.765 |  | -0.1659 | 0.4526 | N |  | 0060089 |
| GA17326 ^b^ | 16 | 1158 | 9 | 0.0030 | 1 | NA | 0.98 |  | -0.0856 | 0.4580 | N |  | 0005215,0060089 |
| GA17328 ^b^ | 14 | 621 | 3 | 0.0000 | 1 | NA | 0.754 |  | 0.2567 | 0.0757 | N |  | — |
| GA17444 ^a^ | 12 | 1071 | 2 | 0.0006 | 1 | NA | 0.64 |  | -0.2095 | 0.4198 | N |  | 0003824,0005488 |
| GA17480 ^b^ | 17 | 630 | 2 | 0.0054 | 1 | NA | 0.356 |  | 0.4659 | 0.0006 | F |  | 0003824,0005488 |
| GA17483 ^b^ | 17 | 903 | 0 | 0.0000 | NA | NA | NA |  | -1.1975 | 0.0000 | M |  | 0003824,0005488 |
| GA17485 ^b^ | 17 | 516 | 0 | 0.0000 | NA | NA | NA |  | 0.4372 | 0.0002 | F |  | — |
| GA17487 ^b^ | 17 | 1059 | 4 | 0.0043 | 1 | NA | 0.354 |  | -0.3154 | 0.0132 | N |  | 0005198,0005488 |
| GA17491 ^a^ | 12 | 1353 | 4 | 0.0034 | 0.287 | NA | 0.654 |  | 2.1491 | 0.0000 | F |  | 0030234 |
| GA17595 ^b^ | 15 | 246 | 0 | 0.0000 | NA | NA | NA |  | 1.9710 | 0.0000 | F |  | 0005198,0005488 |
| GA17601 ^b^ | 17 | 1209 | 9 | 0.0079 | 1 | NA | 0.765 |  | 0.0215 | 0.9827 | N |  | 0005488,0030528 |
| GA17618 ^b^ | 17 | 1149 | 0 | 0.0000 | NA | NA | NA |  | 0.2052 | 0.2256 | N |  | 0030528 |
| GA17623 ^b^ | 16 | 1059 | 0 | 0.0000 | NA | NA | NA |  | -0.0831 | 0.5850 | N |  | 0003824 |
| GA17717 ^a^ | 12 | 1092 | 2 | 0.0012 | 0.132 | NA | 0.77 |  | 1.2018 | 0.0000 | F |  | — |
| GA17765 ^b^ | 17 | 534 | 2 | 0.0021 | 1 | NA | 0.556 |  | 0.5688 | 0.0002 | F |  | — |
| GA17778 ^b^ | 14 | 969 | 3 | 0.0030 | 1 | NA | 0.555 |  | 0.7744 | 0.0000 | F |  | 0005488,0030528 |
| GA17791 ^b^ | 17 | 1230 | 6 | 0.0022 | 1 | NA | 0.362 |  | -0.2533 | 0.0895 | N |  | 0005488,0030528 |
| GA17804 ^b^ | 17 | 861 | 2 | 0.0012 | 1 | NA | 0.235 |  | 0.3540 | 0.0007 | F |  | 0005488 |
| GA17840 ^b^ | 16 | 585 | 1 | 0.0000 | 1 | NA | 0.165 |  | -0.7271 | 0.0000 | M |  | 0003824 |
| GA18060 ^b^ | 17 | 249 | 0 | 0.0000 | NA | NA | NA |  | -0.8372 | 0.0000 | M |  | 0005198,0005488 |
| GA18109 ^b^ | 17 | 678 | 6 | 0.0111 | 1 | NA | 0.145 |  | -0.1981 | 0.3373 | N |  | 0005215 |
| GA18665 ^c^ | 18 | 1071 | 2 | 0.0014 | 1 | NA | 0.143 |  | 1.1467 | 0.0000 | F |  | 0005488,0030528 |
| GA18828 ^b^ | 16 | 900 | 1 | 0.0026 | 1 | NA | 0.465 |  | 1.2458 | 0.0000 | F |  | 0003824 |
| GA19084 ^a^ | 12 | 1179 | 6 | 0.0054 | 0.145 | NA | 0.543 |  | -0.1061 | 0.5573 | N |  | — |
| GA19833 ^b^ | 15 | 1371 | 3 | 0.0005 | 1 | NA | 0.46 |  | 0.7293 | 0.0000 | F |  | 0030528 |
| GA19840 ^a^ | 12 | 1125 | 1 | 0.0006 | 0.163 | NA | 0.423 |  | 0.1016 | 0.7414 | N |  | — |
| GA20863 ^b^ | 17 | 768 | 3 | 0.0031 | **0.017*** | 0.023 | 0.343 |  | -0.0770 | 0.7339 | N |  | 0003824 |
| GA20930 ^c^ | 18 | 717 | 0 | 0.0000 | NA | NA | NA |  | -0.1226 | 0.6737 | N |  | 0003824,0005488,0060089 |
| GA20960 ^b^ | 15 | 915 | 1 | 0.0007 | 1 | NA | 0.765 |  | -0.0740 | 0.6422 | N |  | 0005488,0030528 |
| GA20985 ^b^ | 17 | 534 | 2 | 0.0018 | 1 | NA | 0.278 |  | 0.8422 | 0.0010 | F |  | — |
| GA21006 ^a^ | 12 | 1251 | 5 | 0.0041 | 1 | NA | 0.543 |  | 0.2532 | 0.3246 | N |  | 0003824,0005488 |
| GA21040 ^c^ | 18 | 1233 | 1 | 0.0017 | **0.033*** | 0.045 | 0.432 |  | 0.4615 | 0.0000 | F |  | — |
| GA21074 ^b^ | 15 | 1263 | 9 | 0.0033 | 1 | NA | 0.876 |  | 0.2644 | 0.2002 | N |  | 0003824,0005488,0030234 |
| GA21089 ^b^ | 17 | 1110 | 9 | 0.0050 | 1 | NA | 0.264 |  | -0.3565 | 0.0020 | M |  | 0003824,0005488 |
| GA21092 ^b^ | 17 | 954 | 3 | 0.0005 | 1 | NA | 0.465 |  | -0.1443 | 0.3697 | N |  | 0003824,0005488 |
| GA21185 ^b^ | 17 | 1209 | 8 | 0.0016 | **0.019*** | **0.928*** | **0.009*** |  | 0.6318 | 0.0061 | F |  | — |
| GA21191 ^c^ | 18 | 1605 | 17 | 0.0120 | 1 | NA | 0.133 |  | -0.0542 | 0.7245 | N |  | 0060089 |
| GA21266 ^a^ | 12 | 378 | 5 | 0.0075 | **0.001*** | **0.433*** | **0.011*** |  | 0.1647 | 0.5822 | N |  | 0005198 |
| GA21268 ^a^ | 12 | 111 | 0 | 0.0000 | NA | NA | NA |  | 0.0472 | 0.8664 | N |  | 0005488,0030528 |
| GA21314 ^a^ | 12 | 888 | 1 | 0.0000 | 0.188 | NA | 0.543 |  | 0.3803 | 0.0262 | N |  | 0003824,0005488 |
| GA21323 ^b^ | 17 | 228 | 0 | 0.0000 | NA | NA | NA |  | 0.4000 | 0.0047 | F |  | — |
| GA21338 ^b^ | 17 | 1014 | 3 | 0.0023 | 1 | NA | 0.243 |  | 1.8527 | 0.0000 | F |  | — |
| GA21372 ^b^ | 17 | 1098 | 2 | 0.0009 | 1 | NA | 0.787 |  | 0.3493 | 0.0442 | N |  | 0005488 |
| GA21373 ^b^ | 17 | 1059 | 11 | 0.0071 | **0.002*** | 0.043 | 0.821 |  | -0.1002 | 0.5469 | N |  | 0003824,0005488 |
| GA21446 ^b^ | 17 | 1014 | 4 | 0.0029 | 1 | NA | 0.867 |  | -0.9768 | 0.0000 | M |  | — |
| GA21447 ^b^ | 17 | 1056 | 3 | 0.0038 | 1 | NA | 0.234 |  | 1.7068 | 0.0000 | F |  | 0003824 |
| GA21461 ^a^ | 12 | 1479 | 1 | 0.0005 | 0.302 | NA | 0.098 |  | -1.9555 | 0.0000 | M |  | — |
| GA21467 ^a^ | 12 | 1122 | 9 | 0.0085 | 0.19 | NA | 0.764 |  | -1.1945 | 0.0000 | M |  | 0003824 |
| GA21479 ^c^ | 17 | 966 | 3 | 0.0010 | 0.291 | NA | 0.754 |  | 0.0037 | 0.8964 | N |  | 0005488,0030528 |
| GA21704 ^b^ | 17 | 786 | 1 | 0.0016 | 1 | NA | 0.154 |  | -0.1662 | 0.4376 | N |  | 0003824 |
| GA21718 ^b^ | 17 | 978 | 3 | 0.0020 | 1 | NA | 0.654 |  | 0.3801 | 0.0479 | N |  | 0005488 |
| GA21795 ^c^ | 18 | 1863 | 4 | 0.0011 | 1 | NA | 0.243 |  | 0.4720 | 0.0000 | F |  | 0005488 |
| GA22088 ^b^ | 17 | 1023 | 2 | 0.0010 | 1 | NA | 0.765 |  | 0.2462 | 0.1912 | N |  | 0005488,0030528 |
| GA22092 ^b^ | 16 | 276 | 0 | 0.0000 | NA | NA | NA |  | 0.1837 | 0.3996 | N |  | 0005215 |
| GA24220 ^c^ | 18 | 1356 | 11 | 0.0031 | **0.018*** | **0.561*** | 0.099 |  | 0.0601 | 0.8244 | N |  | — |
| GA24366 ^b^ | 17 | 1116 | 8 | 0.0079 | **0.008*** | **0.949*** | 0.765 |  | -0.4430 | 0.0000 | M |  | 0003824,0005488 |
| GA24366 ^b^ | 17 | 1206 | 11 | 0.0040 | NA | NA | NA |  | -0.4430 | 0.0000 | M |  | 0003824,0005488 |
| GA24434 ^a^ | 12 | 1101 | 6 | 0.0012 | 0.222 | NA | 0.212 |  | 0.3728 | 0.0805 | N |  | 0003824 |
| GA24439 ^c^ | 18 | 936 | 11 | 0.0069 | 0.231 | NA | 0.199 |  | 0.2049 | 0.3511 | N |  | 0005488 |
| GA24492 ^b^ | 16 | 1698 | 3 | 0.0003 | 0.306 | NA | 0.654 |  | -0.0920 | 0.5792 | N |  | 0003824 |
| GA24493 ^b^ | 17 | 636 | 5 | 0.0000 | 0.795 | NA | 0.263 |  | — | — | — |  | — |
| GA24496 ^b^ | 17 | 789 | 5 | 0.0028 | 1 | NA | 0.254 |  | 0.2641 | 0.0119 | N |  | — |
| GA24514 ^a^ | 12 | 414 | 4 | 0.0048 | 1 | NA | 0.199 |  | 0.1647 | 0.5822 | N |  | 0005198 |
| GA24730 ^c^ | 18 | 1200 | 4 | 0.0028 | **0.012*** | **0.773*** | 0.511 |  | 0.4168 | 0.0422 | N |  | 0005488,0030528 |
| GA24823 ^c^ | 18 | 456 | 8 | 0.0131 | 0.065 | NA | 0.543 |  | -0.0688 | 0.7124 | N |  | 0003824 |
| GA24841 ^b^ | 17 | 480 | 7 | 0.0244 | 0.497 | NA | 0.645 |  | -0.1825 | 0.0646 | N |  | 0005488 |
| GA24843 ^b^ | 17 | 153 | 0 | 0.0000 | NA | NA | NA |  | 0.4853 | 0.0005 | F |  | 0005488,0030234,0030528 |
| GA24935 ^b^ | 17 | 1230 | 2 | 0.0008 | 0.887 | NA | 0.645 |  | 0.7146 | 0.0006 | F |  | 0005488 |
| GA24936 ^b^ | 16 | 900 | 0 | 0.0000 | NA | NA | NA |  | -0.3077 | 0.0434 | N |  | 0003824 |
| GA24989 ^a^ | 12 | 246 | 3 | 0.0041 | **0.006*** | **0.432*** | **0.022*** |  | -0.1554 | 0.1343 | N |  | 0003824,0005488 |
| GA25087 ^b^ | 17 | 480 | 1 | 0.0041 | 1 | NA | 0.654 |  | -0.0557 | 0.8181 | N |  | 0003824 |
| GA25088 ^b^ | 12 | 1092 | 1 | 0.0013 | 1 | NA | 0.143 |  | -1.6051 | 0.0000 | M |  | 0003774,0003824,0005198 |
| GA25089 ^b^ | 17 | 294 | 0 | 0.0000 | NA | NA | NA |  | 0.4971 | 0.0001 | F |  | 0005488,0030528 |
| GA26099 ^a^ | 12 | 1179 | 9 | 0.0027 | 1 | NA | 0.654 |  | 0.1863 | 0.2355 | N |  | 0003824 |

^a^ Bachtrog & Andolfatto 2006

^b^ this study

^c^ Bartolome et al. 2006

^d^ Number of synonymous sites studies

^e^ Count of segregating synonymous sites

^f^ The weighted average within-species pairwise synonymous diversity per synonymous site.

^g^ Locus-specific p-value for the CLRT test (Kim & Stephan 2002)

^h^ Locus-specific p-value for the GOF test (Jensen et al. 2005)

^i^ Locus-specific p-value for the CLRT test (Kim & Nielsen 2004)

^j^ Locus-specific female/male expression ratios inferred from *D. pseudoobscura* (Sturgill et al. 2007).

^k^ Locus-specific expression bias (M: male-biased expression; F: female-biased expression, N: non-biased expression.

^l^ Locus-specific gene ontology classes extracted from FlyBase (<http://flybase.bio.indiana.edu/>)

Bachtrog, D and Andolfatto, P, Selection, recombination and demographic history in Drosophila miranda. *Genetics* **174** (4), 2045 (2006).

Bartolomé, C et al., Patterns of selection on synonymous and nonsynonymous variants in Drosophila miranda. *Genetics* **169** (3), 1495-507 (2005).

Jensen, JD et al., Distinguishing between selective sweeps and demography using DNA polymorphism data. *Genetics* **170** (3), 1401 (2005).

Kim, Y and Stephan, W, Detecting a local signature of genetic hitchhiking along a recombining chromosome. *Genetics* **160** (2), 765 (2002).

Kim, Y and Nielsen, R, Linkage disequilibrium as a signature of selective sweeps. *Genetics* **167** (3), 1513 (2004).

Sturgill D, Zhang Y, Parisi M, Oliver B Demasculinization of X chromosome genes in the Drosophila genus. (2007). Nature **450**:238-241.
