# Supplementary material for: Accelerated Adaptive Evolution on a Newly Formed X Chromosome
Source: PLoS Biol. 2009 Apr 14;7(4):e1000082. doi: 10.1371/journal.pbio.1000082 (PMC2672600; doi:10.1371/journal.pbio.1000082)
Supplement: Table S3 — (57 KB DOC) [file pbio.1000082.st003.doc]

**Table S3. Major functional categories of genes located on the ancestral X and the neo-X chromosome.**

| GO Category^a^ | ancestral X^b^ | neo-X^c^ | p-value^d^ |
| --- | --- | --- | --- |
| Antioxidant activity GO:0016209 | 1 gene  (1.1%) | 0 genes  (0.0%) | - |
| Binding GO:0005488 | 65 genes  (71.4%) | 60 genes (58.3%) | 0.7255 |
| Catalytic activity GO:0003824 | 42 genes  (46.2%) | 49 genes (47.6%) | 0.4146 |
| Enzyme regulator activity GO:0030234 | 6 genes  (6.6%) | 4 genes  (3.9%) | - |
| Molecular transducer activity GO:0060089 | 15 genes  (16.5%) | 12 genes (11.7%) | 0.5943 |
| Motor activity GO:0003774 | 8 genes  (8.8%) | 3 genes  (2.9%) | - |
| Structural molecule activity GO:0005198 | 17 genes  (18.7%) | 14 genes (13.6%) | 0.6235 |
| Transcription regulator activity GO:0030528 | 10 genes  (11.0%) | 19 genes (18.4%) | 0.08586 |
| Translation regulator activity GO:0045182 | 4 genes  (4.4%) | 1 gene  (1.0%) | - |

^a^ Gene ontology classes were extracted from FlyBase (<http://flybase.bio.indiana.edu/>). A Chi-squared test reveals no significant heterogeneity in gene count among GO categories between the ancestral X and the neo-X (χ^2^=9.52; d.f.=8; p=0.30).

^b^ 91 genes located on the ancestral X have GO level 2 information (see Supplementary Table 1)

^c^ 103 genes located on the neo-X chromosome have GO level 2 information (see Supplementary Table 2)

^d^P-values for Chi-squared test of equal expected proportions between ancestral X and neo-X by GO category
