# Supplementary material for: Accelerated Adaptive Evolution on a Newly Formed X Chromosome
Source: PLoS Biol. 2009 Apr 14;7(4):e1000082. doi: 10.1371/journal.pbio.1000082 (PMC2672600; doi:10.1371/journal.pbio.1000082)
Supplement: Table S4 — (KB DOC). [file pbio.1000082.st004.doc]

**Table S4. Number of genes located on the ancestral X and the neo-X chromosome with male-biased, female-biased and non-biased expression.**

| Expression Bias^a^ | ancestral X^b^ | neo-X^c^ | P-value^d^ |
| --- | --- | --- | --- |
| unbiased | 74 gene (67%) | 91 genes (61%) | 0.66 |
| Female-biased | 22 genes (20%) | 37 genes (25%) | 0.60 |
| Male-biased | 14 genes (13%) | 21 genes (14%) | 0.75 |

^a^ To classify genes as female-, male- or non-biased, microarray data from *D. pseudoobscura* were used (Sturgill et al. 2007). A Chi-squared test reveals no significant heterogeneity in gene count among sex-biased expression categories between the ancestral X and the neo-X (χ^2^=1.3; d.f.=2; p=0.52).

^b^110 genes located on the ancestral X have information on patterns of sex-biased expression (see Supplementary Table 1)

^c^149 genes located on the neo-X chromosome have information on patterns of sex-biased expression (see Supplementary Table 2)

^d^P-values for Chi-squared test of equal expected proportions between ancestral X and neo-X by sex-biased expression

Sturgill D, Zhang Y, Parisi M, Oliver B Demasculinization of X chromosome genes in the Drosophila genus. (2007). Nature **450**:238-241.
