# Supplementary material for: Accelerated Adaptive Evolution on a Newly Formed X Chromosome
Source: PLoS Biol. 2009 Apr 14;7(4):e1000082. doi: 10.1371/journal.pbio.1000082 (PMC2672600; doi:10.1371/journal.pbio.1000082)
Supplement: Table S5 — (38 KB DOC) [file pbio.1000082.st005.doc]

**Table S5. Origin of *Drosophila miranda* strains used for sequence analysis**

| strain | Geographic origin |
| --- | --- |
| 0101.3 | Port Coquitlam, BC, Canada |
| 0101.4 | Port Coquitlam, BC, Canada |
| 0101.5 | Port Coquitlam, BC, Canada |
| 0101.7 | Port Coquitlam, BC, Canada |
| 0101.9 | Mather, CA, USA |
| MA28 | Mather, CA, USA |
| MA32 | Mather, CA, USA |
| MA03.1 | Mather, CA, USA |
| MA03.2 | Mather, CA, USA |
| MA03.3 | Mather, CA, USA |
| MA03.4 | Mather, CA, USA |
| MA03.5 | Mather, CA, USA |
| MA03.6 | Mather, CA, USA |
| SP138 | Spray, OR, USA |
| SP235 | Spray, OR, USA |
| SP295 | Spray, OR, USA |
| MSH22 | Mt. St. Helena, CA, USA |
| MSH38 | Mt. St. Helena, CA, USA |
