# Supplementary material for: Accelerated Adaptive Evolution on a Newly Formed X Chromosome
Source: PLoS Biol. 2009 Apr 14;7(4):e1000082. doi: 10.1371/journal.pbio.1000082 (PMC2672600; doi:10.1371/journal.pbio.1000082)
Supplement: Table S6 — (43 KB DOC) [file pbio.1000082.st006.doc]

**Table S6. Two-class HKA test**

| Test class | # pooled loci | S | D_xy_ |  | χ^2^(obs) | Mean χ^2^(sim) | Prob χ^2^  (sim≥obs) |
| --- | --- | --- | --- | --- | --- | --- | --- |
|  |  |  |  |  |  |  |  |
| XL+XR | 111 | 608 | 1108.7 | - | - | - | - |
| neo-X | 151 | 414 | 1405.7 | 7.1 | 1.29 | 0.03 | <10^-4^ |
|  |  |  |  |  |  |  |  |

NOTE – Following the approach of Andolfatto (2005) except all polymorphisms are included. Only common (i.e. excluding singletons) polymorphisms were used to estimate parameters θ (for each locus) and T, the species divergence time. Probabilities are based on 10^4^ coalescent simulations with no intragenic recombination. Note that the standard χ^2^ approximation to significance with 1 d.f. (i.e. χ^2^ =3.84) does not hold because the calculation of the χ^2^ statistic (Hudson et al. 1987) assumes that there is no recombination between loci within the pooled test classes.

Andolfatto P. Adaptive evolution of non-coding DNA in Drosophila (2005). Nature **437**:1149-1152.

Hudson RR, Kreitman M, Aguadé M. A test of neutral molecular evolution based on nucleotide data (1987). Genetics **116**:153-159.
