# Supplementary material for: Accelerated Adaptive Evolution on a Newly Formed X Chromosome
Source: PLoS Biol. 2009 Apr 14;7(4):e1000082. doi: 10.1371/journal.pbio.1000082 (PMC2672600; doi:10.1371/journal.pbio.1000082)
Supplement: Table S7 — (22 KB DOC) [file pbio.1000082.st007.doc]

Table S7. Numbers of loci showing evidence for recent adaptive evolution.

|  | CLR test^a^ | ω_max_ test^b^ |
| --- | --- | --- |
| X | 0 | 0 |
| neo-X | 14 | 6 |

^a^The number of loci showing evidence for adaptive evolution at a false discovery rate of 20% (Storey 2002) using the CLR test.

^b^The number of loci showing evidence for adaptive evolution at a false discovery rate of 20% (Storey 2002) using the ω_max_ test.

Storey JD. (2002) A direct approach to false discovery rates. *Journal of the Royal*

*Statistical Society, Series B*, **64**: 479-498.
